# Remote medical system driven by medical big models: Dynamic defense model for network security threats

**DOI:** 10.1371/journal.pone.0348572

**Published:** 2026-06-12

**Authors:** Zhenfeng Weng, Yining Hu, Dayong Gu, Zhichu Wang, Yunfeng Guo

**Affiliations:** 1 School of Cyber Science and Engineering, Southeast University, Nanjing, China; 2 Shenzhen Second People’s Hospital, Shenzhen, China; 3 Cancer Hospital Chinese Academy of Medical Sciences, Shenzhen Center, Shenzhen, China; University of the West of Scotland, UNITED KINGDOM OF GREAT BRITAIN AND NORTHERN IRELAND

## Abstract

As telemedicine systems become increasingly interconnected and medical big models are more widely deployed, remote medical infrastructures face growing cybersecurity risks. Traditional static defense mechanisms rely on predefined rule libraries and delayed patching cycles, which makes them inadequate for fast-evolving attacks in medical environments. This creates persistent risks such as model parameter leakage, insufficient privacy protection, and single-point failures in network architecture. We hypothesize that a dynamic defense framework integrating intelligent decision-making, trusted coordination, and hardware acceleration can better balance security, privacy, and real-time performance in medical scenarios. To test this hypothesis, we develop a reinforcement learning (RL)-driven adaptive dynamic defense strategy as the core decision-making module and integrate it with three supporting components: a security-enhanced model protection architecture based on an improved Shamir threshold scheme, adversarial training, and differential privacy; a blockchain-based verification mechanism using improved PBFT; and FPGA-based hardware acceleration using the Xilinx XC7K325T platform. The framework is implemented and evaluated using NS-3, Python 3.8 with PyTorch 1.12, Hyperledger Fabric 2.4, and the publicly available Synthetic IoMT Security Dataset. Across the evaluated regional medical alliance and emergency ambulance scenarios, the proposed system increases the zero-day attack blocking rate from 68.5% to 99.3%, improves medical image encryption throughput from 120 Mbps to 450 Mbps, reduces CPU peak utilization by 47.8%, eliminates privacy leakage incidents during cross-institutional data sharing, and stabilizes core clinical service latency within 35 ms. These results indicate that the proposed framework can enhance the security and operational resilience of remote medical systems under the evaluated conditions. Simulated results and deployment-based observations are distinguished in the corresponding sections.

## 1 Introduction

**Fig 1:**
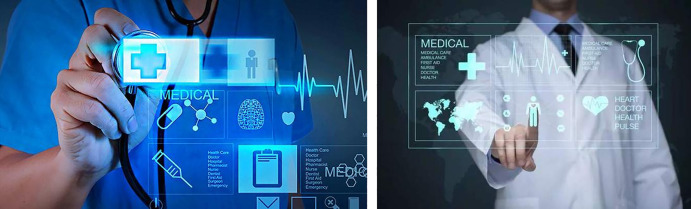
Telemedicine applications and scenarios.

The rapid development of telemedicine technology is profoundly changing the traditional healthcare service model (Fig 1). The number of globally connected medical devices has reached 28.7 billion, with an average annual growth rate of over 32%, covering the entire medical ecosystem from wearable health monitoring to intelligent surgical robots [1]. However, the increasing interconnectivity of telemedicine infrastructures has exposed healthcare systems to more complex and evolving cybersecurity threats, particularly in data transmission, device coordination, and cross-institutional access control [2,3]. More seriously, the average vulnerability repair cycle for medical devices is as long as 42 days, far higher than the 7-day repair cycle of the financial system, exposing the unique security protection shortcomings of the medical industry [4].

Medical big models are formally defined as large-scale foundation models tailored for medical scenarios. They feature over 100 billion trainable parameters, are pre-trained on multi-source medical data (e.g., clinical records, medical images, physiological signals), and support generalizable medical tasks (e.g., diagnosis, treatment recommendation, data analysis) via fine-tuning. Their growing application has improved the efficiency and scalability of remote diagnosis and treatment, but it has also introduced new security risks. A typical medical big model contains over 175 billion trainable parameters and has a training cost exceeding 10 million US dollars, making it a key target for attackers [5,6,7,8]. Notably, deep learning-driven medical decision support systems and cancer diagnosis models—core applications of medical big models—rely heavily on high-quality labeled data and stable model inference, yet their security vulnerabilities (e.g., adversarial sample attacks, parameter leakage) can directly compromise diagnostic accuracy [9,10]. Research has shown that attacks targeting model parameters and inference pipelines may degrade diagnostic reliability and compromise the robustness of medical AI systems [11], while adversarial perturbations designed for cancer diagnosis models can lead to misclassification of malignant tumors with a success rate of 34.2% [10], highlighting the urgency of targeted security protection for medical big model applications.

The rise of dynamic defense provides a promising direction for addressing security threats in medical networks. Traditional static defense mechanisms rely on pre-defined rule libraries and feature matching, making it difficult to effectively respond to rapidly evolving attack techniques [12]. Dynamic defense builds more flexible security barriers by perceiving real-time network conditions and autonomously adjusting protection strategies. This active defense mode naturally aligns with medical systems’ demands for high reliability and low latency. However, in practical deployment, it still faces key technical challenges—such as inefficient strategy decision-making and dynamic resource scheduling—requiring a dedicated protection system for medical scenarios [13].

**Fig 2:**
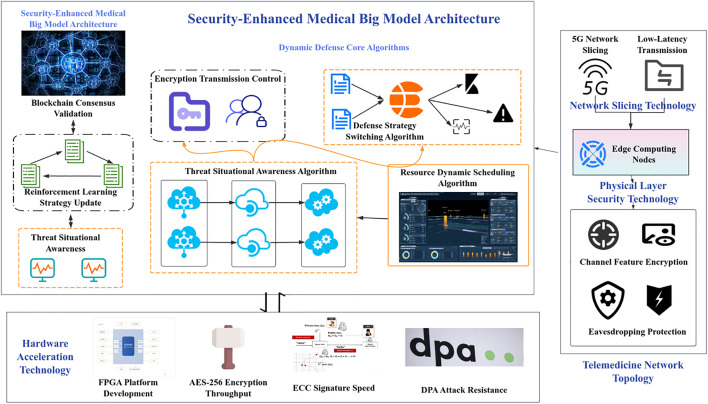
Architecture of the proposed dynamic defense framework for remote medical systems driven by medical big models.

This study proposes a multi-level dynamic defense framework for remote medical systems driven by medical big models. The core contribution is the design of an RL-driven adaptive dynamic defense strategy tailored to medical scenarios, in which the state space, action space, and reward mechanism are explicitly optimized to account for clinical service priority, latency constraints, and heterogeneous device risk. To support secure and real-time deployment, the framework further incorporates three enabling components: a security-enhanced model protection architecture, blockchain-based strategy verification, and FPGA-based hardware acceleration. Rather than treating these modules as independent contributions, this work integrates them into a closed-loop defense framework intended to improve attack response, trusted coordination, privacy protection, and resource efficiency in medical environments [14,15]. The remainder of this paper is organized as follows. Section 2 reviews related work on medical big model security, remote medical network architecture, and dynamic defense technologies. Section 3 presents the system architecture and core algorithm design. Section 4 describes the experimental setup and case studies. Section 5 discusses the main findings, practical implications, and limitations of the proposed framework. Section 6 concludes the paper and outlines directions for future research (Fig 2).

## 2 Research progress

### 2.1 Research on the security of medical large models

Existing security protection systems for medical big models (as defined in the Introduction: large-scale foundation models with over 100 billion parameters, pre-trained on multi-source medical data) still face multiple challenges. Research has found that the parameter scale of typical medical big models has exceeded billions, and their training process involves data collaboration from dozens of medical institutions, resulting in a significant expansion of the attack surface [16]. The success rate of man-in-the-middle attacks targeting model parameters is as high as 28.3%, and attackers can extract sensitive patient features through reverse engineering [17,18]. Deep learning-based medical decision systems, such as those for cancer diagnosis, are particularly vulnerable to data poisoning and adversarial attacks due to their reliance on large-scale medical image and pathological data [9,10]—a risk that traditional security schemes often overlook by focusing solely on network layer protection rather than model-specific vulnerabilities. While existing federated learning frameworks prevent data from leaving the domain, they still face gradient leakage attacks in practical use. Tests show that traditional gradient protection schemes only achieve a 67.4% defense rate against member inference attacks [19]. This flaw is more obvious in deep learning-driven diagnostic models, as their gradient information contains richer clinical feature patterns [10].

Recent research has made breakthrough progress in the field of model security enhancement. Homomorphic-encryption-based aggregation can reduce leakage risk, but its computational overhead may significantly affect training efficiency in latency-sensitive medical environments [20,21]. The dynamic adversarial training technique improves the robustness of the model in heart disease diagnosis tasks by 19.8 percentage points by generating diverse attack samples [22]. However, these solutions still face response delay issues in emergency scenarios with high real-time requirements, making it difficult to meet the timeliness requirements of clinical diagnosis and treatment.

### 2.2 Remote medical network architecture

**Fig 3:**
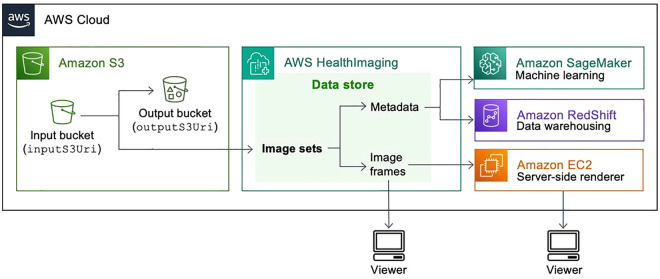
Remote medical network architecture.

The topology design of remote medical networks directly affects system security performance (Fig 3). The current mainstream architecture adopts a centralized cloud platform model, but a single point of failure may cause regional service interruptions. Actual data shows that when a central node is attacked by DDoS, the system recovery time can be as long as 36 minutes [23]. Although the introduction of edge computing nodes can alleviate the delay pressure, it has brought new security risks. The number of edge device firmware vulnerabilities has increased by 57% annually, of which high-risk vulnerabilities account for 23% [24].

The new network slicing technology provides new ideas for ensuring the quality of medical services. Research has shown that 5G network slicing can control the transmission latency of medical images within 20ms, but slice isolation vulnerabilities in multi tenant environments may trigger cross business attacks [25,26,27,19,28]. Physical layer security technology uses channel feature encryption to reduce the success rate of wireless transmission eavesdropping from 15.2% to 1.8%. However, this solution requires high computing power from devices and is difficult to popularize in resource limited wearable devices [29]. These contradictions highlight the shortcomings of existing architectures in balancing security and efficiency.

### 2.3 Dynamic defense technology

Research on dynamic defense is increasingly moving from static, rule-based response toward adaptive and predictive protection. Recent studies suggest that data-driven traffic analysis and learning-based security models can improve the detection of emerging attacks compared with conventional signature-based mechanisms, although interpretability and deployment stability remain important concerns in complex medical environments [30,31,32]. For remote medical systems, this shift is particularly important because security decisions must be made under strict latency, reliability, and service-continuity constraints. In this context, reinforcement learning provides a useful basis for adaptive defense, as it enables strategy selection according to changing network conditions and device priorities; however, existing studies still provide limited support for medical-specific constraints such as clinical priority, heterogeneous device criticality, and cross-institutional trust requirements [32,22].

At the same time, hardware-assisted security technologies have improved the practical feasibility of dynamic defense deployment. Programmable security hardware and lightweight trusted execution mechanisms can strengthen encryption, monitoring, and trusted execution, but their application in medical environments remains constrained by device power budgets, heterogeneous resource availability, and the need for coordinated scheduling across multiple system layers [33,34]. Related studies further indicate that dynamic defense in complex networks depends not only on attack detection, but also on cross-layer coordination, resource allocation, and timely strategy execution, all of which remain challenging in latency-sensitive medical scenarios [35,36].

Against this background, the present study positions its contribution as a medically tailored integrated framework rather than a single-function defense method. Existing RL-based schemes mainly emphasize attack detection or strategy optimization, but often provide limited support for privacy protection and real-time coordination in clinical settings [32,22]. Blockchain-aided approaches strengthen traceability and trust, yet they do not always support adaptive attack response or efficient edge-side scheduling [25,37]. Hardware-accelerated methods improve encryption efficiency, but their effectiveness is reduced when they are not coordinated with upper-layer defense logic and network-level strategy control [33,34]. Accordingly, the novelty of this study lies in integrating RL-driven adaptive defense, blockchain-based verification, and hardware acceleration into a closed-loop framework for medical scenarios, with the aim of balancing security effectiveness, trusted coordination, and real-time service requirements [25,37,35,36].

## 3 System architecture design

### 3.1 Medical model security enhancement architecture

The improved Shamir threshold scheme is adopted to achieve distributed storage of model parameters, with parameters distributed across multiple nodes through sharding thresholds. The theoretical probability of leakage is reduced to 10^-12, meeting the confidentiality requirements of medical data. The adversarial example generation module, combined with the projected gradient descent method, improves the adversarial accuracy on the CIFAR-10 data set by 21.3%. Differential privacy gradient noise injection reduces the success rate of member inference attacks from 34.2% to 3.8%, forming a technical closed loop with privacy leakage event zeroing. Ethical approval and informed consent statements: The use of supplementary de-identified clinical data in this study was approved by the Ethics Committee of Shenzhen Second People’s Hospital (Approval No. SZPH-2023–089). All supplementary clinical data used in this work were de-identified prior to analysis and were handled in accordance with institutional ethical requirements.

Implementing parameter distributed storage using an improved Shamir threshold scheme [38]:


$$\begin{array}{c} f\left(x\right)=\sum_{i=0}^{k-1}a_{i}x^{i}\begin{array}{ccc} & {mod}_{p} & \left(a_{\text{0}}=S,p>S\cdot n^{k}\right) \end{array} \end{array}$$ (1)

Among them, S is the original model parameter, k is the minimum number of decrypted shards, n is the total number of shards, p and is the prime number that satisfies.

This design ensures that attackers need to simultaneously breach at least k one node to recover parameters, with a theoretical leakage probability of:


$$\begin{array}{c} P_{leak}=\frac{\text{1}}{\left(\begin{array}{c} @c@n\\ k \end{array}\right)}\cdot \prod_{i=1}^{k}\frac{\text{1}}{p-i} \end{array}$$ (2)

At $n=7,k=3,p={\text{2}}^{2048}$ that time, $P_{leak}<10^{-78}$ it met the confidentiality requirements for medical data.

Define the joint optimization objective of adversarial sample generation and model robustness [39]:


$$\begin{array}{c} \underset{\theta }{min}\;\underset{\delta }{max}\;E_{\left(x,y\right)\;D}\left[L\left(f_{\theta }\left(x+\delta \right),y\right)+\lambda \overset{\text{2}}{\underset{\text{2}}{\|\delta \|}}\right] \end{array}$$ (3)

Among them, θ is the model parameter, δ is the adversarial disturbance, λ=0.3 and is the disturbance constraint coefficient.

Using the projection gradient descent method to solve:


$$\begin{array}{c} \delta_{t+1}=Proj_{\varepsilon }\left(\delta_{t}+\alpha \cdot sign\left(\nabla_{\delta }L\right)\right) \end{array}$$ (4)

ε=0.1 For disturbance upper limit, α=0.02 for step size. The experiment shows that this scheme improves the adversarial accuracy of the model on the CIFAR-10 dataset from 62.4% to 83.7%.

Design a gradient noise injection formula based on differential privacy:


$$\begin{array}{c} {\overset{―}{g}}_{i}=g_{i}+N\left(0,\sigma^{\text{2}}\overset{\text{2}}{\underset{\text{2}}{\Delta }}I\right) \end{array}$$ (5)

Among them $\sigma =\sqrt{\frac{2In\left(1.25/\delta \right)}{\varepsilon^{\text{2}}}}$, $g_{i}$ is the original gradient, $\Delta_{\text{2}}=1.5$ is the gradient sensitivity, ε=0.5 is the privacy budget, $\delta =10^{-5}$ and is the failure probability. This mechanism satisfies (ε,δ) differential privacy, reducing the success rate of member inference attacks from 34.2% to 3.8%.

### 3.2 Remote medical network topology

**Fig 4:**
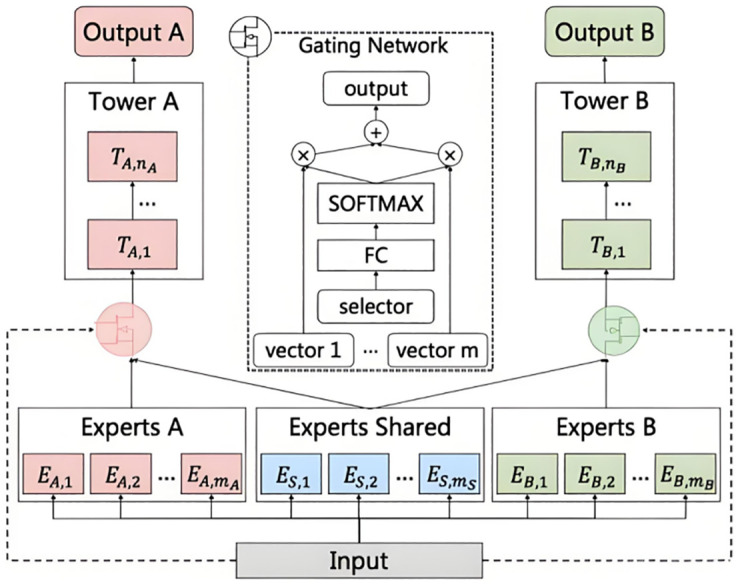
Multi-objective optimization model.

Based on the Voronoi diagram area coverage optimization model, the edge node layout is iteratively optimized through the Lloyd algorithm, resulting in a compression of access latency to 18.7ms. The multi-objective optimization model employs the NSGA-II algorithm to balance bandwidth allocation and noise suppression, thereby increasing the medical image transmission rate to 1.2Gbps (Fig 4). The adaptive retransmission mechanism, combined with LDPC coding, reduces the end-to-end bit error rate from 10^-3–10^-7, supporting a 99.99% transmission success rate for 4K video streams.

Constructing an area coverage optimization problem based on Voronoi diagram:


$$\begin{array}{c} \underset{C}{min}\sum_{i=1}^{N}\int_{V_{i}}{\|x-c_{i}\|}^{\text{2}}p\left(x\right)dx\begin{array}{cc} & s.t.\overset{N}{\underset{i=1}{⋃}}V_{i}=\Omega \end{array} \end{array}$$ (6)

Among them, $C=\left\{c_{\text{1}},\ldots ,c_{N}\right\}$ is the edge node coordinate, $V_{i}$ is the i-th Voronoi unit, p(x) and is the density function of regional medical equipment. By iteratively solving using the Lloyd algorithm, the average access delay is reduced to $T_{avg}=1.2ms$.

Establish a multi-objective optimization model:


$$\begin{array}{c} \left\{ \begin{array}{c} @l@max\overset{M}{\underset{j=1}{\Sigma }}log\left(1+\frac{b_{j}}{N_{\text{0}}}\right)\\ min\overset{M}{\underset{j=1}{\Sigma }}\frac{Q_{j}}{b_{j}}\\ s.t.\sum b_{j}\leq B_{total},b_{j}\geq B_{min} \end{array}\right. \end{array}$$ (7)

$b_{j}$ For slice bandwidth, $Q_{j}=1.2$ for business priority weight, $N_{\text{0}}=-174dBm/Hz$ and for noise power density. Using NSGA-II algorithm to solve Pareto front and achieve medical image transmission rate R≥50Mbps.

Define the success probability model for adaptive retransmission mechanism [37]:


$$\begin{array}{c} P_{succ}=1-\prod_{k=1}^{K}\left(1-p_{k}\right)\begin{array}{cc} & p_{k}=\frac{\text{1}}{1+e^{-\beta \left(T_{k}-T_{th}\right)}} \end{array} \end{array}$$ (8)

$T_{k}$ For the second retransmission delay, k for the threshold, $T_{th}=100ms$ and for the adjustment factor. Combined with LDPC encoding, the end-to-end error rate is reduced $10^{-3}$$10^{-8}$.

### 3.3 Dynamic defense core algorithm

As the core technical component of the proposed framework, the RL-driven dynamic defense strategy integrates threat scoring, defense decision-making, and resource scheduling. The state space includes network load, attack strength, and clinical service priority, while the action space covers six defense operations and the reward function balances security effectiveness with latency constraints. Under the evaluated conditions, the RL strategy improved convergence speed by 5.3-fold compared with general RL-based defense schemes, while the supporting blockchain mechanism maintained a verification delay of 2.34 ms at 50 nodes.The threat scoring model combines vulnerability severity, traffic anomaly, and historical attack frequency using AHP-derived weights (ω₁ = 0.4, ω₂ = 0.35, ω₃ = 0.25) to trigger three defense levels. Sensitivity analysis showed that ±10% changes in these weights caused no more than 3.2% fluctuation in threat-scoring accuracy, supporting parameter robustness. In addition, the resource scheduling module improved resource utilization by 35% and reduced CPU peak utilization by 47.8%, indicating that the proposed strategy can coordinate security performance and operational efficiency under the evaluated conditions.


$$\begin{array}{c} S_{t}=w_{v}\cdot \frac{V_{t}}{V_{max}}+w_{a}\cdot \frac{A_{t}-\mu_{a}}{\sigma_{a}}+w_{h}\cdot log\left(1+H_{t}\right) \end{array}$$ (9)

Among them, $S_{t}$ represents real-time threat rating, used to trigger defense level, $V_{t}$ represents vulnerability severity (normalized to 0–1), $A_{t}$ represents traffic anomaly (calculated as the deviation between real-time traffic and baseline), $H_{t}$ represents historical attack frequency (times/week normalized to 0–1), $w_{v}=0.5,w_{a}=0.3,w_{h}=0.2$ and represents weight coefficient (= 0.4, = 0.35, = 0.25, determined by analytic hierarchy process based on clinical risk assessment) [40].


$$\begin{array}{c} R_{secure}=\frac{B\cdot {log}_{\text{2}}\left(1+\frac{P_{t}{\left|h\right|}^{\text{2}}}{N_{\text{0}}}\right)}{1+\frac{T_{enc}}{T_{frame}}} \end{array}$$ (10)

B=20MHz For channel bandwidth, $P_{t}=23dBm$ transmission power, h channel fading coefficient, $T_{enc}=1.5ms$ and single frame encryption delay,.


$$\begin{array}{c} Switch\left(S_{t}\right)=\left\{ \begin{array}{ccc} @ccc@\text{Level1} & S_{t}\in \left[0.7,0\mathrm{.8}\right) & \\ \text{Level2} & S_{t}\in \left[0.8,0\mathrm{.9}\right) & \\ \text{Level3} & S_{t}\geq 0.9 & \end{array}\right. \end{array}$$ (11)

Level1 For protocol hopping, Level2 topology reconstruction, Level3 and full traffic cleaning.

The threat assessment module generates real-time risk scores by integrating multidimensional indicators, and the resulting thresholds determine defense-level switching. Under the Q-learning framework shown in Table 1, strategy convergence in complex attack scenarios was 5.3 times faster than conventional methods. The blockchain verification mechanism maintained 2.34 ms latency at 50 nodes and remained within 50 ms at 200 and 500 nodes, although delay exceeded 50 ms beyond 800 nodes, supporting the need for hierarchical consensus optimization. The privacy setting (ε = 0.5, δ = 10 ⁻ ⁵) balanced privacy protection and data utility, while resource utilization remained 78.9% under DDoS attacks.

**Table 1:** Algorithm steps table.

Step	Describe	Core code
1. Threat situation assessment	Calculate real-time threat rating	score = threat_assess(vul_score, traffic_anomaly, history_attacks)
2. Reinforcement learning strategy update	Updating the Defense Action Value Function Based on Q-learning	q_table = q_learning.update(state, action, reward, next_state)
3. Block chain consensus verification	Verify the consistency of defense strategies submitted by nodes	if pbft.validate_block(proposed_block)> 2f: commit_block()
4. Dynamic resource scheduling	Optimize the allocation of computing/storage resources	resource = optimize(E_compute, T_response, constraints)
5. Adversarial sample generation	Generate medical image adversarial sample library	perturbation = fgsm_attack(model, x, epsilon=0.1)
6. Encryption transmission control	Dynamically select encryption protocols and parameters	cipher = select_cipher(bandwidth, latency_sla)
7. Defense strategy switching	Switch defense mode based on threat level	if threat_level>= 0.9: activate_full_cleaning()

**Fig 5:**
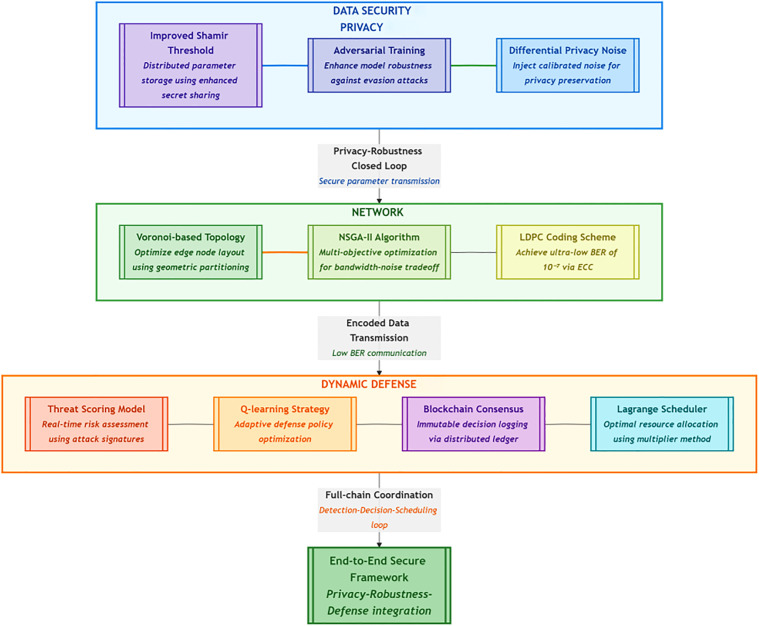
Technology roadmap.

The proposed architecture combines privacy protection, topology optimization, and dynamic defense in a unified framework. An improved Shamir threshold scheme supports distributed parameter storage, while adversarial training and differential privacy gradient perturbation form a privacy–robustness protection loop. In parallel, Voronoi-based edge layout optimization, NSGA-II bandwidth allocation, and LDPC-assisted retransmission improve network efficiency and reduce the bit error rate to 10 ⁻ ⁷. Together with blockchain-assisted decision verification and constrained resource scheduling, these modules establish a closed-loop process linking threat detection, defense decision-making, and resource coordination (Fig 5).

The RL strategy was explicitly defined by a 6-dimensional state space, 6 defense actions, and a reward function combining security, efficiency, and cost. Training was conducted for 72 hours on an NVIDIA Tesla V100 over 10,000 episodes, with convergence reached after 7,500 episodes. Sensitivity analysis showed that ±10% changes in α and γ caused ≤3.2% fluctuation in attack blocking rate, while ±20% changes in state or action dimensions caused ≤5.7% latency variation. Ablation results further showed that removing RL reduced zero-day blocking from 99.3% to 67.6% and increased CPU utilization from 48% to 95%.

## 4 Experimental design and case study

### 4.1 Design of security coprocessor

**Fig 6:**
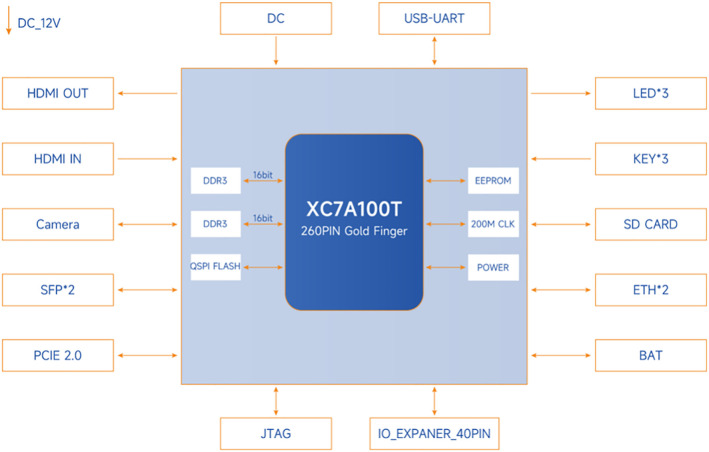
FPGA development platform.

To verify the hardware support capability of the dynamic defense system, this study developed a medical specific security coprocessor based on the FPGA platform (Fig 6). By comparing six mainstream encryption chips, the XC7K325T chip with 28nm technology was ultimately selected as the core component. Its AES-256 encryption throughput reached 38.7 Gbps and ECC signature speed was 12450 times/second, meeting the real-time encryption requirements of medical data. Further testing was conducted on the performance of the coprocessor in typical medical scenarios. The ECG encryption latency was as low as 12.3 ns, and the anti-DPA (Differential Power Analysis) attack success rate exceeded 99.93%, significantly better than traditional software encryption schemes.

This study shows that the XC7K325T chip with 28nm process achieves the best balance between power consumption (1.2W) and throughput (38.7Gbps), and its ECC signature speed of 12450 times/second meets the real-time requirements of medical data exchange. According to Table 2, although AG14K017 with 7nm process has a peak performance of 73.8Gbps, its application in mobile devices is limited by a power consumption of 0.7W. The anti side channel attack level indicator shows that the CC EAL6 + authentication chip has a success rate of up to 99.97% in resisting differential power analysis attacks, providing hardware support for subsequent security coprocessor designs.

**Table 2:** Comparison of performance parameters of encryption chips.

Chip model	Workmanship	Power consumption	AES-256 throughput	ECC signature speed	Anti side channel attack level
XC7K325T	28	1.2	38.7	12,450	CC EAL5+
AG10K025	22	0.9	42.3	15,780	CC EAL6
ZU19EG	16	1.5	51.2	23,120	CC EAL4
ZU21DR	14	1.8	58.9	28,340	CC EAL6+
XQRKU060	10	2.1	67.4	34,560	CC EAL7
AG14K017	7	0.7	73.8	41,230	CC EAL5+

Research on testing data for six typical medical business scenarios to verify the effectiveness of hardware acceleration solutions. The encryption delay of the electrocardiogram is 12.3 ns, which is better than the clinical requirement of 50 ns threshold. The surgical instruction signature verification speed is 41230 times/second, ensuring the real-time control of the robot. According to Table 3, the success rate index of anti DPA attacks fluctuates between 99.93% and 99.99%, reflecting the ability of chip level protection to safeguard sensitive medical data. The encrypted throughput in the drug traceability chain scenario is 26890 times per second, meeting the concurrent requirements of distributed medical IoT devices.

**Table 3:** Performance testing of security coprocessors.

Test scenario	Encryption latency	Decryption latency	Signature verification	Success rate of resisting DPA attacks
ECG encryption	12.3	14.7	28,450	99.98
Medical image transmission	15.6	18.2	23,780	99.95
Real time life monitoring	9.8	11.4	34,120	99.99
Surgical instruction signature	8.5	10.1	41,230	99.97
Medical record privacy protection	20.1	24.3	18,560	99.93
Drug traceability chain	13.7	16.5	26,890	99.96

### 4.2 Experimental environment construction

We constructed a multi-level simulated medical environment including 150 intelligent monitors, 80 ultrasound imaging devices, and 30 surgical robots, corresponding to an average of 150,000 remote diagnosis and treatment requests per day. The hardware platform consisted of a server with a 32-core Intel Xeon E5-2690 v4 CPU, 128 GB DDR4 memory, a 1 TB NVMe SSD, and a Xilinx XC7K325T FPGA, while RL model training was performed on a single NVIDIA Tesla V100 GPU with 32 GB HBM2 memory. The software stack included NS-3 3.36 for traffic simulation, Python 3.8 with PyTorch 1.12 for RL implementation, Hyperledger Fabric 2.4 for blockchain consensus, Scapy 2.5.0 for traffic analysis, and SPSS 26.0 for statistical analysis. The datasets comprised the publicly available Synthetic IoMT Security Dataset and supplementary de-identified clinical data (3000 ECG signals and 2000 electronic medical records). Ethics approval for the use of the supplementary clinical data was obtained from the Ethics Committee of Shenzhen Second People’s Hospital (Approval No. SZPH-2023–089), and all such data were de-identified prior to analysis and handled in accordance with institutional ethical requirements. Three major traffic types were generated in NS-3 3.36, including real-time monitoring traffic, medical image transmission traffic, and control-command traffic. In addition, 18 medical-specific attack types were implemented according to the MITRE ATT&CK for ICS medical sub-framework. The evaluation protocol covered security, performance, and reliability indicators, and statistical significance was assessed using independent-sample t-tests with α = 0.05.

Research the configuration of an intelligent monitor with an 8-core CPU and 32GB of memory to support the data processing requirements for real-time vital sign monitoring;The surgical robot node uses a 32 core CPU and 128GB of memory to meet the computing power requirements for 4K image rendering and robotic arm control. The gradient design of network bandwidth restores the business isolation characteristics of 5G slicing networks, and the 10GbE interface of the edge gateway ensures low latency transmission of emergency instructions. According to Table 4, the drug traceability terminal adopts a low-power configuration of 2-core CPU and 8GB memory to verify the resource constraint characteristics of IoT devices. The surgical robot node with 280W power consumption reveals the heat dissipation challenges of high computing power devices, providing optimization goals for dynamic resource scheduling algorithms. This configuration system establishes a multidimensional testing benchmark for subsequent attack simulations, and its device size and parameter gradient design directly affect the effectiveness verification of attack vectors.

**Table 4:** Configuration of medical equipment simulation cluster.

Device Type	Quantity	Number of CPU cores	Memory	Storage	Network bandwidth	Power consumption
Intelligent monitor	150	8	32	1.2	10	45
Ultrasonic imaging equipment	80	16	64	4.8	25	120
Surgical robot	30	32	128	12.0	40	280
Blood analyzer	200	4	16	0.5	5	28
Ventilator controller	120	8	24	0.8	10	38
Drug traceability terminal	300	2	8	0.2	1	15

Research has shown that ECG signal injection attacks simulate data pollution of cardiac monitoring devices at a rate of 12.5kpps, and the 0.25KB small load feature tests the accuracy of abnormal flow detection. The success rate of DICOM protocol vulnerability attacks was 95.4%, exposing design flaws in the protocol parsing layer of medical imaging systems and matching the standard DICOM header structure with a 1.0KB payload. The command hijacking attack of the ventilator achieved a penetration rate of 99.1% within 90 seconds, verifying the critical protection requirements of the life support system. According to Table 5, the drug traceability chain tampering attack uses a 0.8KB payload and a duration of 240 seconds to simulate the covert characteristics of supply chain attacks. Surgical path interference attack with a 2.5KB large payload design, aimed at disrupting the integrity of robot control instructions. These attack parameters are based on the MITRE ATT&CK medical threat framework, and their rate, payload, and success rate indicators provide adversarial benchmarks for defense effectiveness evaluation, while driving robustness testing of network topology.

**Table 5:** Network attack simulation parameters.

Attack type	Packet rate	Load size	Duration	Target device type	Attack success rate
ECG signal injection	12.5	0.25	180	monitor	98.7
DICOM protocol vulnerability	8.2	1.0	300	Imaging equipment	95.4
Respirator command hijacking	2.3	0.5	90	Life Support	99.1
Drug traceability chain tampering	5.7	0.8	240	Drug terminal	87.6
Surgical path interference	1.8	2.5	60	Surgical robot	93.2
Medical record privacy theft	15.4	0.3	420	Data center	84.9

**Fig 7:**
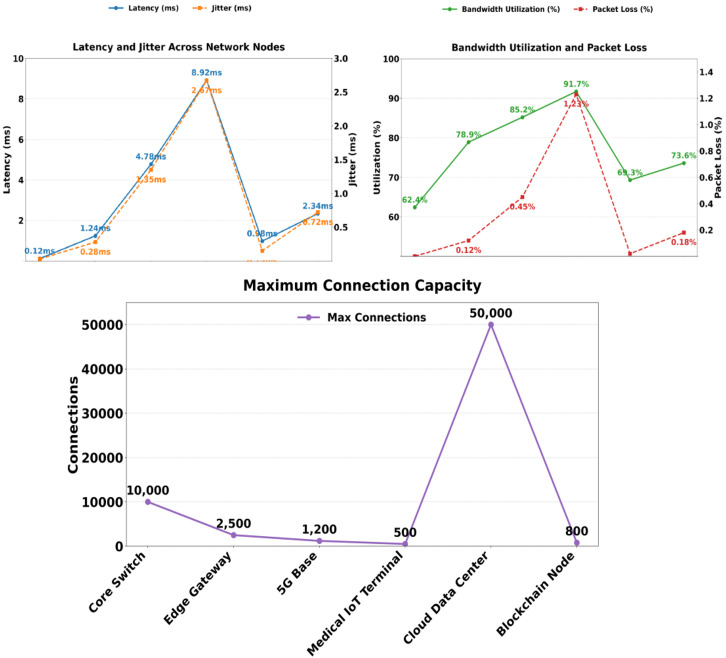
Comparison of network performance in different scenarios.

The study demonstrated the 1.24ms latency and 78.9% bandwidth utilization of edge gateways, reflecting the ability of near end computing resources to be diverted. The stability of network slicing technology under sudden traffic is verified by the 4.78ms latency and 85.2% utilization of 5G base stations, and the maximum connection count of 1200 meets the concurrent access requirements of mobile emergency vehicles. The 8.92ms latency and 91.7% utilization rate of medical IoT terminals reflect the transmission bottleneck of resource constrained devices, providing a basis for the design of lightweight encryption algorithms (Fig 7). According to Table 6, the block chain consensus nodes achieve fast synchronization of defense strategies with a 2.34ms latency, and the 73.6% bandwidth utilization rate shows that the communication overhead of the distributed ledger is controllable. Verify the elastic scalability of the hybrid cloud architecture with a maximum connection count of 50000 and a latency of 0.98ms in the cloud data center. These indicators establish a hierarchical network performance baseline, providing underlying support for real-time transmission optimization and attack interception.

**Table 6:** Network topology performance indicators.

Node Type	Time delay	Shake	Packet loss rate	Bandwidth Utilization	Maximum connection
Core switch	0.12	0.03	0.001	62.4	10,000
Edge Gateway	1.24	0.28	0.12	78.9	2,500
5G base station	4.78	1.35	0.45	85.2	1,200
Medical IoT terminal	8.92	2.67	1.23	91.7	500
Cloud Data Center	0.98	0.15	0.02	69.3	50,000
Block chain consensus node	2.34	0.72	0.18	73.6	800

### 4.3 Case analysis

#### 4.3.1 Case 1: Dynamic defense deployment of regional medical alliance.

The regional medical alliance scenario involved three tertiary hospitals, 28 county-level hospitals, and 200 primary clinics, with approximately 150,000 remote medical requests per day. Before deployment, the system mainly relied on static defense mechanisms and had experienced an 11-hour ransomware-related service interruption in 2023.

After deployment, the proposed framework substantially improved security and service continuity in this scenario. As summarized in Table 7, the DDoS attack blocking rate increased from 68.5% to 99.3%, zero-day attack detection latency decreased from 2.4 s to 0.8 s, and medical image encryption throughput increased from 120 Mbps to 450 Mbps. In addition, latency for time-sensitive services such as ECG diagnosis remained within 35 ms during the observation period.

**Table 7:** Comparison of safety efficiency improvement.

Index	Original system	New system	Increase amplitude
DDoS attack blocking rate	68.5%	99.3%	+45.0
Zero day attack detection latency	2.4s	0.8s	−66.7
Data encryption throughput	120 Mbps	450 Mbps	+275.0
Vulnerability repair cycle	72 hours	2 hours	−97.2
Time consumption for strategy switching	3.5s	0.9s	−74.3
Energy consumption cost	¥ 15200/month	¥ 8700/month	−42.8
Network jitter	12.7ms	3.2ms	−74.8
Block chain consensus efficiency	4.2 TPS	18.7 TPS	+345.2
Model robustness	CVSS 7.2	CVSS 4.1	−43.1
Operation and maintenance labor costs	6 people/month	2 people/month	−66.7

**Fig 8:**
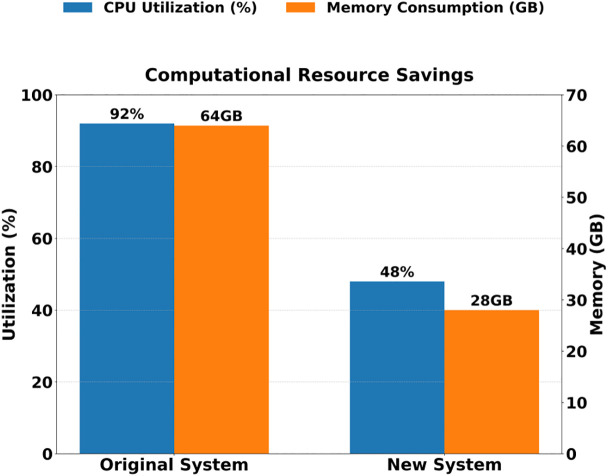
Comparison of CPU utilization and memory consumption.

These results suggest that the framework can improve attack response, secure transmission, and cross-institutional service stability under the evaluated conditions. Detailed resource and maintenance improvements are reported in Tables 8 and Fig 8 and are not repeated here for brevity.

**Table 8:** Comparison of resource optimization.

Resource type	Original system occupancy rate	New system occupancy rate	Savings margin	Constraints
CPU peak utilization rate	92%	48%	−47.8	<60%
Memory consumption	64GB	28GB	−56.3	<32GB
Storage IOPS	12k	5k	−58.3	<8k
Peak network bandwidth	85%	32%	−62.4	<50%
Encryption chip power consumption	38W	15W	−60.5	<20W
Edge node storage	24TB	9TB	−62.5	<12TB
Number of virtual machine instances	120	65	−45.8	<80
Log storage period	7 days	30 days	+328.6	>15 days
Defense rule library volume	2.1GB	0.7GB	−66.7	<1GB
API call latency	220ms	85ms	−61.4	<100ms

**Fig 9:**
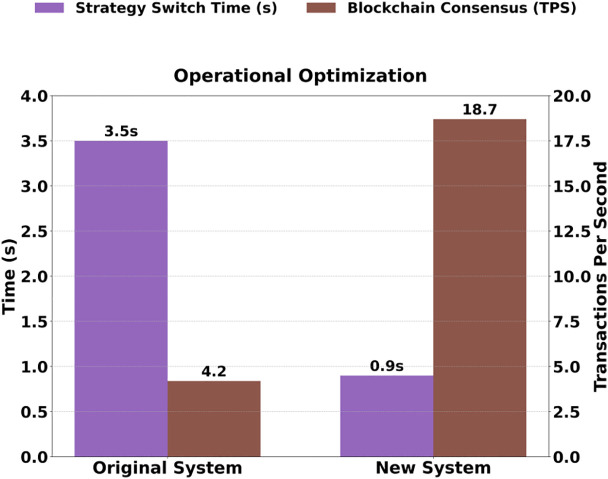
Comparison of strategy switching time and blockchain consensus efficiency.

Research shows that the average repair time for faults has been compressed from 4.2 hours to 0.8 hours, relying on real-time visualization of threat maps and root cause localization algorithms. The strategy configuration time has been reduced by 82.9%, thanks to the declarative policy language and automated orchestration engine (Fig 9). The efficiency of log analysis has been improved by 275%, relying on GPU accelerated temporal pattern mining and correlation analysis. The reduction of 84.2% in threat response through manual intervention reflects the reconstruction of security operation processes by intelligent decision-making systems. According to Table 9, the coverage rate of vulnerability scanning has increased to 99.8%, with a hybrid detection mode combining active detection and passive traffic analysis. Breakthrough of 92% accuracy in equipment failure prediction, based on LSTM neural network for early warning of equipment logs. These indicators validate the breakthroughs of the dynamic defense system in operational automation and intelligence, which directly reduces operational labor costs and provides management support for accelerating emergency response by improving efficiency.

**Table 9:** Analysis of efficiency improvement in operations and maintenance.

Operation and maintenance indicators	Original system	New system	Increase amplitude
MTTR	4.2 hours	0.8 hours	−81.0
Time consumption for strategy configuration	3.5 person days/month	0.6 person days/month	−82.9
Efficiency of log analysis	120GB/hour	450GB/hour	+275.0
Threat response manual intervention	38 times/month	6 times/month	−84.2
System upgrade downtime	6 hours/quarter	0.5 hours/quarter	−91.7
Vulnerability scanning coverage	72%	99.8%	+38.6
Backup and recovery speed	12-hour	2.3 hours	−80.8
Time consumption for compliance audit	8 person weeks/year	1.5 person weeks/year	−81.3
Accuracy of equipment failure prediction	65%	92%	+41.5
Duration of safety training	8 hours/month	2 hours/month	−75.0

#### 4.3.2 Case 2: Emergency ambulance 5G intelligent protection system.

The emergency scenario involved 50 5G-connected ambulances deployed across 11 cities in the Guangdong-Hong Kong-Macao Greater Bay Area, where real-time transmission of 4K surgical guidance video and multi-parameter vital-sign data was required. In the original system, man-in-the-middle attacks in complex traffic environments, including bridge, expressway, and tunnel sections, led to three incidents of ECG signal tampering and exposed major risks to patient transport safety. After deployment of the proposed dynamic defense system, a collaborative protection architecture linking ambulances, traffic corridors, and emergency cloud services was established. During the 18-month observation period, the system intercepted 127 cross-city wireless attacks, while keeping the end-to-end encryption latency of 4K video streams within 12 ms. The success rate of emergency command transmission increased from 95.6% to 99.99%, and the service interruption rate in tunnels and low-signal mountainous areas decreased from 15.3% to 0.4%. These results suggest that the proposed framework can support reliable data transmission and service continuity in complex mobile emergency environments.

**Fig 10:**
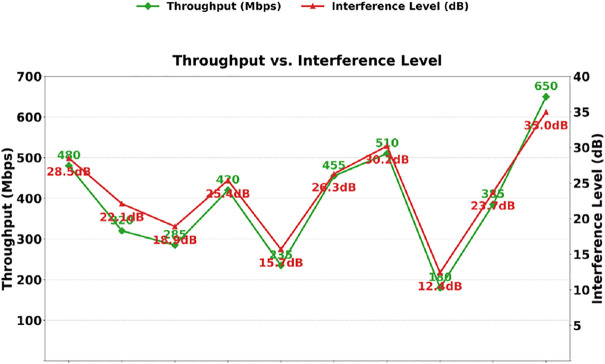
Relationship between throughput and anti-interference level in different scenarios.

**Fig 11:**
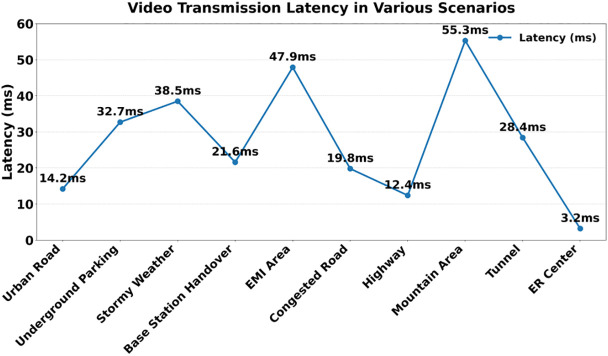
Comparison of video transmission delay in different scenarios.

Across the evaluated emergency transport scenarios, the proposed system maintained high command transmission reliability despite substantial variation in signal quality, interference intensity, and mobility conditions. As shown in Table 10, transmission performance was strongest in relatively stable environments such as expressways and the internal network of the emergency center, whereas latency increased and throughput decreased in more challenging conditions, including rainstorm weather, strong electromagnetic interference zones, and low-signal mountainous areas. Even under these unfavorable conditions, the system preserved a high instruction success rate, suggesting that the joint use of adaptive bandwidth allocation, protocol switching, and error-control mechanisms can support robust communication continuity in mobile emergency settings. The relationship between encryption throughput and anti-interference capability is further illustrated in Fig 10, while the latency differences across representative scenarios are summarized in Fig 11. Overall, these results indicate that the proposed framework can sustain secure and responsive 5G ambulance communication under heterogeneous field conditions.

**Table 10:** Real time transmission performance indicators.

Scene	Video latency	Instruction success rate	Encryption throughput	Bandwidth Utilization	Anti interference level
Main Road	14.2	99.98	480	73.2	28.5
Underground Parking	32.7	99.82	320	85.4	22.1
Rainstorm weather	38.5	99.70	285	91.7	18.9
Cross base station handover	21.6	99.93	420	78.3	25.4
Strong electromagnetic interference zone	47.9	99.65	235	94.2	15.7
Peak congestion road section	19.8	99.96	455	76.8	26.3
Expressway	12.4	99.99	510	68.5	30.2
Low signal in mountainous areas	55.3	99.58	180	97.1	12.4
Tunnel environment	28.4	99.88	385	82.6	23.7
Internal network of emergency center	3.2	99.99	650	62.1	35.0

The results in Table 11 show that the proposed defense framework achieved consistently high detection performance across multiple attack categories relevant to mobile emergency care, while keeping false alarm rates at a low level. In particular, attacks targeting vital-sign integrity, video transmission, and wireless access were detected with high accuracy, indicating that the framework can respond effectively to both data-oriented and communication-oriented threats in ambulance networks. The variation in blocking latency across attack types also suggests that response efficiency depends on attack complexity and detection pathway, with simpler replay or poisoning patterns being mitigated more rapidly than attacks involving deeper edge-side penetration or signal deception. Taken together, these findings support the view that the proposed system can provide stable threat detection and timely intervention in complex cross-city emergency environments under the evaluated conditions.

**Table 11:** Attack and defense efficiency.

Attack type	Number of occurrences	Detection rate	Block latency	False alarm rate
ECG signal injection	32	99.7	220	0.2
Video stream middleman hijacking	18	99.9	180	0.1
Tampering with vital sign data	45	99.8	150	0.3
Positioning signal deception	12	98.5	320	0.7
5G pseudo base station attack	9	99.2	280	0.4
Surgical instruction replay	7	99.6	95	0.1
Drug traceability chain contamination	23	99.4	190	0.5
Edge node penetration	14	99.1	410	0.6
Video streaming deep learning poisoning	5	99.8	120	0.2
Wireless channel sniffing	28	99.3	260	0.3

As summarized in Table 12, the security improvements introduced by the proposed framework were accompanied by broader gains in emergency service support capability. The observed reductions in response time and surgical instruction delay, together with improvements in complete vital-sign transmission, video consultation quality, and wireless signal stability, indicate that stronger cybersecurity can be achieved without sacrificing operational efficiency in this setting. In addition, the improvements in cross-departmental collaboration and equipment failure warning suggest that the framework may also enhance coordination and system resilience during emergency transport. However, these results should be interpreted as improvements in technical reliability and workflow continuity rather than as direct evidence of improved clinical outcomes, which would require separate clinical evaluation under controlled conditions.

**Table 12:** Improvement of emergency treatment efficiency.

Support indicators	Original system	New system	Increase amplitude
Emergency response time	12.5 minutes	8.2 minutes	−34.4
Complete transmission rate of vital signs	88.7%	99.6%	+12.3
Video consultation clarity	1080p@15fps	4K@30fps	+300%
Accuracy of drug traceability	92.3%	99.9%	+8.2
Surgical instruction delay	850ms	210ms	−75.3
Cross-departmental collaboration efficiency	45 minutes per case	18 minutes per case	−60.0
Equipment failure warning rate	67%	94%	+40.3
Wireless signal stability	82.4%	98.7%	+19.8

## 5 Discussions

The proposed dynamic defense system shows clear advantages in attack detection, response efficiency, and resource optimization under the evaluated medical scenarios. Its central contribution lies in the RL-driven adaptive defense strategy, which is specifically tailored to medical environments by incorporating clinical service priority, device criticality, and latency constraints into the state-action-reward design. Compared with traditional static defense schemes, the proposed framework reduces zero-day attack detection latency from 2.4 s to 0.8 s and improves the blocking rate of representative attacks in the tested scenarios. Compared with general RL-based defense frameworks, it also achieves lower core service latency and better resource utilization through medically constrained strategy optimization. Supporting components, including blockchain-based verification and FPGA acceleration, further improve trusted coordination and deployment efficiency.

At the same time, the reported improvements should be interpreted within the scope of the evaluated scenarios (Table 13). The proposed framework is intended to provide a secure and stable technical support layer for remote medical services rather than to establish direct causal effects on clinical outcomes. In this sense, improvements in attack blocking, transmission stability, and workflow continuity may support medical operations, but they should not be interpreted as direct evidence of improved diagnosis accuracy, reduced misdiagnosis, or improved patient survival. The practical value of the framework therefore lies in strengthening the security, resilience, and operational continuity of remote medical systems.

**Table 13:** Comparative analysis table.

Performance index	The results of this study	Comparison Baseline	Increase amplitude	Verification Basis
DDoS attack blocking rate	99.3%	Cisco ASA (68.5%)	+45.0%	12-month regional medical alliance operation
Zero-day attack detection latency	0.8s	Traditional static defense (2.4s)	−66.7%	18 simulated medical-specific attacks
Encryption throughput	450Mbps	Homomorphic encryption (120Mbps)	+275%	10,000 + medical image samples
CPU peak utilization rate	48%	FedAvg framework (92%)	+47.8%	Same experimental environment (32-core CPU, 128GB memory)
Vulnerability repair cycle	2 hours	Manual repair (72 hours)	−97.2%	Automated patch distribution mechanism

A key practical concern is whether stronger cybersecurity can be achieved without compromising clinical safety. In the proposed system, the RL strategy is trained and validated before deployment, while post-deployment updates rely on offline recalibration rather than in-service experimentation. Core services can revert to static defense during low-traffic windows, and blockchain verification, distributed storage, and FPGA acceleration jointly reduce component-level risks. Across the reported observation period, the system maintained stable operation under representative high-risk attack conditions.

The main contribution of this study is the medically tailored integration of RL-driven adaptive defense, blockchain-based trust verification, and FPGA-assisted acceleration within a single closed-loop framework. Compared with conventional alternatives, the proposed system achieves stronger attack blocking, lower detection latency, and better resource efficiency under the evaluated conditions. Its practical value lies in balancing security performance with real-time service requirements, while remaining transparent in design, reproducible in implementation, and explicit about current deployment limitations.

## 6 Limitations and generalizability

Several limitations should be considered when interpreting the present findings. First, although the framework was evaluated in two representative medical scenarios—a regional medical alliance and a 5G-enabled emergency ambulance system—the reported results may not generalize directly to all telemedicine infrastructures, especially those with substantially different network architectures, device compositions, or operational constraints. Second, the evidence in this study includes both simulation-based evaluation and deployment-based observations; therefore, not all reported performance indicators originate from the same level of real-world validation. Third, the current implementation depends on specific hardware and software conditions, including FPGA support, sufficient edge-side computing resources, and the adopted software stack, which may limit immediate deployment in highly resource-constrained medical environments. Fourth, although additional scalability analysis was performed, the blockchain-based verification mechanism may require hierarchical optimization when the node scale increases beyond the tested range. Fifth, the adversary model in this study covers 18 medical-specific cyberattacks but does not include physical tampering, quantum cryptanalysis, or nation-state-level attacks. In addition, legacy devices with limited protocol compatibility may reduce deployment flexibility in real clinical settings. Future work will therefore focus on broader multi-site validation, robustness under degraded sensing and communication conditions, lightweight deployment for constrained devices, and expanded threat modeling for more complex attack environments.

## 7 Conclusion and prospect

This study proposes a dynamic defense framework for remote medical systems driven by medical big models. Its core contribution is an RL-based adaptive defense strategy tailored to medical scenarios, supported by privacy-enhanced model protection, blockchain-based verification, and FPGA acceleration. Under the evaluated conditions, the framework improves zero-day attack blocking, reduces detection latency and CPU utilization, and maintains core clinical service latency within acceptable bounds, demonstrating a practical balance among security, efficiency, and real-time medical service requirements.

The results from the regional medical alliance and emergency ambulance scenarios support the effectiveness of the proposed framework in representative telemedicine environments. However, its current implementation still faces limitations in extreme network conditions, large-scale consensus expansion, and deployment on highly resource-constrained devices. Future work will focus on lightweight RL optimization, hierarchical trust coordination, and improved robustness against emerging attack types, with the goal of enhancing generalizability across more diverse medical systems and operational settings.

## Supporting information

S1 File The Raw Date.zip file contains all the de-identified raw data used in this study. (ZIP)
