# Supplementary material for: Remote medical system driven by medical big models: Dynamic defense model for network security threats
Source: PLoS One. 2026 Jun 12;21(6):e0348572. doi: 10.1371/journal.pone.0348572 (PMC13262880; doi:10.1371/journal.pone.0348572)
Supplement: S1 File — (ZIP) [file pone.0348572.s001.zip › Raw Date/README.md.docx]

# Weixian Paper Cuttings Cultural Field Modeling

This repository implements the field model and boundary deduction algorithm from the paper:

Multi-scale Evaluation and Marginal Deduction Research on the Diffusion of Weixian Paper Cuttings Culture Based on the Field Model

---

## Project Overview

The project models the cultural diffusion of Weixian Paper Cuttings using:

1. Field Strength Calculation: Aggregates 6 subfields (inheritance, media, marketing, education, academic, exhibition) with dynamic decay.

2. Boundary Deduction: Uses the CRCM algorithm to detect core (217km) and diffusion (812km) boundaries.

3. Multi-scale Evaluation: Analyzes diffusion across micro (0-50km), meso (50-500km), and macro (>500km) scales.

---

## Repository Structure

| File Name | Purpose | Paper Section |

|-----------|---------|---------------|

| `1_preprocessing.py` | Load/clean raw data, split into core/peripheral areas | Section2.1 |

| `2_field_strength.py` | Compute weighted field strength with dynamic decay | Section2.2 |

| `3_boundary_deduction.py` | Detect boundaries using CRCM algorithm | Section2.3 |

| `4_multi_scale_evaluation.py` | Evaluate diffusion across scales | Section4.2 |

---
