# Supplementary material for: Remote medical system driven by medical big models: Dynamic defense model for network security threats
Source: PLoS One. 2026 Jun 12;21(6):e0348572. doi: 10.1371/journal.pone.0348572 (PMC13262880; doi:10.1371/journal.pone.0348572)
Supplement: Attachment — Submitted filename: reviwers revison.docx [file pone.0348572.s002.docx]

The topic is timely and relevant to secure remote medical systems, and the manuscript presents an ambitious integrated framework combining dynamic defense, intelligent decision-making, and system-level optimization. However, the current version needs revision to strengthen clarity, rigor, and reproducibility.

1. Clearly distinguish what is new compared with existing dynamic defense/zero-trust/security orchestration frameworks, and summarize contributions explicitly.

2. Define all baselines (“original system” and literature comparisons) with equivalent tuning and matched conditions to avoid unfair comparisons.

3. Provide complete experimental details (datasets/traffic generation, attack implementation, parameters, hardware/software specs, and evaluation protocol).

4. Formalize attacker capabilities, assumptions, and in-scope/out-of-scope attacks to align security claims with evidence.

5. Specify state/action/reward design, training procedure, convergence behavior, and include sensitivity analysis and ablations isolating RL’s effect.

6. Quantify the individual contribution of each module (threat scoring, RL scheduling, blockchain logging/consensus, hardware acceleration).

7. Explain why blockchain is necessary, report consensus/security assumptions, and quantify end-to-end overhead (latency, bandwidth, storage).

8. Use normalized metrics (throughput-per-watt, latency) and report true end-to-end system gains including offload overhead.

9. Clarify whether results are simulated or real deployment; avoid causal clinical outcome statements without a controlled study design.

10. Ensure the data availability statement is consistent and actionable; improve writing clarity, terminology consistency, and report variability (mean±std/CI) for key results.
