# Supplementary material for: Remote medical system driven by medical big models: Dynamic defense model for network security threats
Source: PLoS One. 2026 Jun 12;21(6):e0348572. doi: 10.1371/journal.pone.0348572 (PMC13262880; doi:10.1371/journal.pone.0348572)
Supplement: Attachment — Submitted filename: Response to Reviewers.docx [file pone.0348572.s004.docx]

**Reviewer #1**

The manuscript presents a comprehensive and technically detailed study on a dynamic defense framework for remote medical systems driven by large medical models. The topic is timely and highly relevant given the increasing reliance on telemedicine and the growing security threats faced by interconnected medical infrastructures. Overall, the work demonstrates strong technical depth, clear motivation, and significant practical relevance, and it makes a valuable contribution to the fields of medical network security and intelligent protection systems.

The proposed multi-level dynamic defense architecture is well-structured and integrates advanced techniques such as reinforcement learning–based strategy optimization, blockchain consensus mechanisms, adaptive network protection, and hardware acceleration. The system design is coherent, and the closed-loop coordination between threat detection, defense decision-making, and resource scheduling is convincingly presented. The experimental evaluation is extensive, covering both simulated environments and real-world case studies, and the reported performance improvements (e.g., attack blocking rate, latency reduction, resource utilization, and system robustness) are impressive and well supported by quantitative results.

The manuscript is generally well organized, and the figures, tables, and case analyses help illustrate the effectiveness and applicability of the proposed approach in realistic medical scenarios, including regional medical alliances and emergency ambulance systems. The discussion and conclusion appropriately summarize the contributions and outline meaningful directions for future work.

1. Clarity and language: Some sections would benefit from minor English-language polishing to improve readability and fluency. In particular, simplifying long sentences and ensuring consistent terminology (e.g., “medical big model,” “large medical model”) would enhance clarity.

Response: Thank you for your suggestion. We have polished the entire English text, simplified long sentences, and unified terminology (changing all "large medical model" to "medical big model") to enhance readability and fluency. The modifications have been made in key chapters such as the abstract, introduction, research progress, discussion, and other sections.

2. Methodological clarification: While the technical framework is rich, brief intuitive explanations (in addition to mathematical formulations) for key components such as the threat scoring model, reinforcement learning strategy updates, and blockchain consensus integration would help readers from a broader audience better understand the design choices.

Response: Thank you for your attention. We have supplemented the intuitive explanations (such as analogies like "security sentry" and "doctor adjusting treatment plan") for the threat scoring model, RL strategy update, and blockchain consensus integration to assist non-professional readers in understanding. The modification location is in Section 3.3, Dynamic Defense Core Algorithm (lines 299-401), where 1-2 sentences of life-oriented analogies are added after the mathematical formulas.

3. Reproducibility and comparison: The experimental section is thorough, but adding a short discussion on reproducibility (e.g., parameter sensitivity, computational overhead, or deployment constraints) and clarifying how baseline methods were selected would further improve transparency.

Response: Thank you for your reminder. We have added a discussion on repeatability at the end of Section 4.3 in Chapter 4, explaining the parameter sensitivity, computational overhead, and the basis for baseline selection (based on scenario matching and functional equivalence principles). The modification is located at the end of Section 4.3 (lines 774-797), where we have added baseline selection logic and an analysis of the impact of parameter fluctuations.

“In the emergency ambulance scenario, the holistic computational and energy overhead of all defense components (RL strategy, blockchain consensus, FPGA encryption) is quantified: the on-board edge controller (8-core CPU) has 9.5% CPU utilization (0.76 cores occupied) and 5.8% memory overhead (1.856GB of 32GB memory); energy overhead is 3.7W (vs. 3.2W baseline without defense), compatible with on-board medical equipment’s low-power requirements (power budget ≤5W). Normalized metrics and true end-to-end gains (including offload overhead) are as follows: ① Throughput-per-watt: FPGA acceleration achieves 375 Mbps/W (450 Mbps ÷ 1.2W), 8.3× higher than software encryption (45 Mbps/W) and 2.1× higher than general-purpose hardware acceleration (178 Mbps/W); ② Latency: End-to-end clinical latency is 35±4.2 ms, with FPGA offload adding only 2.3±0.6 ms (vs. 15±2.1 ms for software offload); ③ Comprehensive gains: Compared with non-offloaded systems, it improves throughput-per-watt by 275%, reduces end-to-end latency by 47.8%, and cuts CPU overhead by 42.3% (from 92% to 48% peak utilization) after accounting for FPGA-CPU communication overhead. To enhance experimental transparency and reproducibility, key details are clarified: Core parameters (threat scoring weights: 0.4/0.35/0.25; RL learning rate: 0.1; blockchain nodes: 50) are stable, with ±10% adjustments leading to ≤3.2% fluctuation in attack blocking rate; cumulative system overhead is ≤5.5% CPU utilization and 896KB memory, requiring edge nodes with ≥8-core CPU, 32GB memory, and FPGA compatibility, with software relying on Python 3.8, PyTorch 1.12, and Hyperledger Fabric 2.4 (source code to be publicly available on GitHub); three baselines (Cisco ASA, FedAvg + gradient masking, Cisco Firepower) are selected for fair comparison, matching the proposed system in scenario, core functions, and verifiability.”

4. Limitations: Including a short paragraph explicitly discussing limitations (e.g., scalability to much larger networks, dependency on specialized hardware, or assumptions about network conditions) would provide a more balanced perspective.

I did not identify any concerns regarding dual publication, research ethics, or publication ethics. Overall, this is a strong manuscript that requires only minor revisions before publication, and I recommend it positively after the authors address the points above.

Response: Thank you for your suggestion. We will explicitly discuss the limitations of the system in the conclusion section, including large-scale network scalability, dependence on dedicated hardware, and assumptions about network conditions. Location for modification: Conclusion and Outlook (lines 901-910), add a new paragraph to elaborate on the limitations and potential directions for improvement.

“Validation in regional medical alliance and emergency ambulance scenarios confirms that the system stabilizes core clinical service latency within 35ms, achieves a 99.3% zero-day attack blocking rate, and maintains robust performance under degraded network quality and fluctuating device loads. While limitations exist in extreme network environments and lightweight wearable device deployment, the framework provides a replicable paradigm for the integration of intelligent defense technologies and medical scenarios. Future research will focus on lightweight optimization of the RL strategy, hierarchical consensus expansion, and defense against emerging threats such as AI-generated adversarial attacks, to further expand the system’s generalizability and adaptability to diverse medical contexts.”

**Reviewer #2**

1. Scope and contribution are not well-defined:

The paper attempts to address too many layers simultaneously such as medical AI, network security, reinforcement learning based defense, blockchain consensus, and FPGA acceleration, without clearly identifying a single primary research contribution. As a result, the manuscript reads more like a conceptual system proposal or white paper than a focused research study. For publication, the authors should substantially narrow the scope and clearly articulate one core technical contribution, with other components treated strictly as background or supporting context.

Response: Thank you for your criticism and correction. We have focused on the core contribution, clearly taking "Medical Customized RL-Driven Dynamic Defense Framework" as the core, with other technologies (blockchain, FPGA) as support.

1. Modifications have been made in the abstract “This study develops a reinforcement learning (RL)-driven adaptive dynamic defense strategy as the core, integrated with three supporting technologies: ① security-enhanced model architecture (improved Shamir threshold scheme + adversarial training + differential privacy), ② blockchain-based consensus verification (improved PBFT), and ③ FPGA hardware acceleration (Xilinx XC7K325T).” (lines 21-26);

2. Introduction “This article proposes a multi-level dynamic defense framework for remote medical systems driven by medical big models, with the core technical contribution being the design and optimization of an RL-driven adaptive dynamic defense strategy tailored to medical scenarios. The supporting technologies (security-enhanced model architecture, blockchain consensus, FPGA acceleration) are designed to address the unique constraints of medical systems (e.g., low latency, high reliability, privacy protection) and fully release the potential of the core RL strategy.” (lines 95-101);

3. Research progress “This study’s core innovation lies in customizing an RL-driven defense strategy for medical scenarios: by optimizing the state-action space design (incorporating clinical service priority) and reward function (balancing security effect and business efficiency), it solves the problem that general RL defense schemes are difficult to apply directly to medical systems.” (lines 192-197);

4. Discussion “Notably, security metrics are associated with clinical service quality but do not directly affect clinical outcomes, as the system only provides a secure and stable technical foundation rather than direct outcome improvements. Compared with general RL-based defense frameworks, its tailored state-action space and reward function control the average latency of key businesses (e.g., ECG diagnosis) within 35ms (40% lower than general schemes), while the RL-driven dynamic scheduling algorithm reduces CPU peak utilization by 47.8% and memory consumption by 56.3% compared with the FedAvg framework. Supporting technologies further enhance practicality: FPGA-based hardware acceleration achieves 450Mbps medical image encryption throughput (275% higher than homomorphic encryption) with 99.95% side-channel attack resistance; the blockchain consensus mechanism improves cross-institutional data sharing audit efficiency by 18 times. The holistic computational and energy overhead of the full-stack defense (RL+blockchain+FPGA) is strictly controlled, compatible with resource-constrained medical environments” (lines 807-821), highlighting the core innovation and clarifying the positioning of each technology.

2. Claims substantially exceed the supporting evidence

The manuscript reports large performance gains across multiple dimensions (e.g: DDoS blocking rates, zero-day detection latency, vulnerability repair cycle time, encryption throughput) and further links these results to improvements in clinical outcomes such as misdiagnosis rate and patient survival. These are very strong claims. However, the paper does not provide sufficient experimental design details, baselines, or statistical analysis to justify them.

Response: Thank you for your rigorous comments. We have removed the causal statements between safety indicators and clinical outcomes (survival rate, misdiagnosis rate), emphasizing only the supportive role of technology in clinical processes, and supplemented with experimental design details and statistical analysis. Modifications are made in the abstract (lines 11-41), Section 4.3 Case Analysis (lines 762-773), and Discussion (lines 808-852), with revised statements and supplemented t-test results.

3. Critical details required for replication are missing or underspecified such as datasets and traffic generation methods, attack models and validation, reinforcement learning formulation (state, action, reward, training regime), baseline systems under identical conditions, and evaluation protocols.

Response: Thank you for your correction. We have supplemented the experimental details in Section 4.2, including dataset sources, traffic generation methods, attack model verification, RL formula details, baseline configuration, and evaluation protocol. Modification location: Section 4.2 Experimental Environment Construction (lines 520-563).

“We constructed a simulated test environment encompassing multi-level medical equipment, including 150 intelligent monitors, 80 ultrasound imaging devices, and 30 surgical robots, simulating an average of 150,000 daily remote diagnosis and treatment requests. To ensure experimental reproducibility and transparency, key details are specified as follows: The hardware configuration includes a server equipped with a 32-core Intel Xeon E5-2690 v4 CPU, 128GB DDR4 2400MHz memory, 1TB NVMe SSD, and a Xilinx XC7K325T FPGA for hardware acceleration, while RL model training relies on a single NVIDIA Tesla V100 GPU with 32GB HBM2 memory; the software stack comprises NS-3 3.36 for traffic simulation, Python 3.8 + PyTorch 1.12 for RL model implementation, Hyperledger Fabric 2.4 for blockchain consensus, Scapy 2.5.0 for traffic analysis, and SPSS 26.0 for statistical analysis. The datasets used include the publicly available Synthetic IoMT Security Dataset (https://github.com/WangMCoder2/ZhenfengWengdata/tree/main) with 1 million+ network flows, attack labels, and train/validation/test splits, as well as supplementary desensitized clinical data (3000 ECG signals, 2000 electronic medical records) available upon request (approved by Shenzhen Second People's Hospital Ethics Committee, No. SZPH-2023-089); traffic simulation is conducted via NS-3 3.36, generating three core traffic types—real-time monitoring (250Hz sampling, 128B packets), medical image transmission (DICOM format, 512KB frames, 15fps), and control commands (64B packets, 100Hz frequency)—calibrated by the MITRE ATT&CK 2023 medical threat report. For attack design and implementation, 18 medical-specific attacks mapped to the MITRE ATT&CK for ICS medical sub-framework (including ECG signal injection, DICOM protocol exploitation, and surgical instruction hijacking) are implemented via Scapy 2.5.0, with parameters (packet rate 1.8-15.4 kpps, duration 60-420s) calibrated to real breach data from 32 healthcare institutions (e.g., ECG injection at 12.5 kpps, DICOM vulnerability attack success rate 95.4%) and verified by "functional verification (Simbionic Surgical Simulator) + traffic feature analysis (Python Scapy library)". The formalized adversary model defines capabilities (can launch data tampering, protocol exploitation, DDoS, and control hijacking; accesses public medical protocols (DICOM 3.0, HL7 FHIR) and partial device interfaces; cannot bypass FPGA-based AES-256 encryption or forge blockchain signatures), assumptions (acts maliciously to disrupt services/steal data/tamper with instructions; targets high-value assets such as surgical robots and ventilators; operates within ≤15kpps bandwidth constraints), and scope (in-scope: 18 medical-specific attacks; out-of-scope: physical tampering, quantum cryptanalysis, and nation-state-level attacks). The evaluation protocol covers security indicators (attack blocking rate, detection latency, false alarm rate), performance indicators (clinical latency, resource utilization), and reliability indicators (business interruption rate, data integrity), with statistical analysis performed via independent sample t-test (α=0.05) using SPSS 26.0; network topology tests confirm a core switch latency of 0.12 ms and 5G base station bandwidth utilization of 85.2%, validating system stability under high load. The RL model undergoes 72 hours of training (10,000 episodes) on the NVIDIA Tesla V100, while attack simulation and performance testing run continuously for 30 days to ensure result stability.”

**Reviewer #3**

1.Clarification of “Medical Big Models” Concept

The manuscript repeatedly uses the term medical big models, but a precise and formal definition is missing. It is unclear whether this refers to large-scale foundation models, task-specific deep learning models, or federated medical AI systems. A clearer conceptual definition, preferably in the Introduction, would improve readability and avoid ambiguity.

Response: Thank you for your rigorous suggestions. We have supplemented the formal definition of the medical large model in the introduction, clarifying it as "a large-scale basic model for medical scenarios, containing ≥100 billion trainable parameters, pre-trained with multi-source medical data, and supporting generalized medical tasks." Modification location: Introduction, adding a clear definition of the term when it is first mentioned to avoid ambiguity.

“Medical big models are formally defined as large-scale foundation models tailored for medical scenarios. They feature over 100 billion trainable parameters, are pre-trained on multi-source medical data (e.g., clinical records, medical images, physiological signals), and support generalizable medical tasks (e.g., diagnosis, treatment recommendation, data analysis) via fine-tuning.” (see lines 62-66).

2.Novelty Compared to Existing Dynamic Defense Frameworks

While the proposed system integrates reinforcement learning, blockchain, and hardware acceleration, the manuscript does not clearly articulate how this integration differs fundamentally from existing dynamic defense or adaptive security frameworks. A dedicated subsection comparing the proposed approach with closely related works would strengthen the novelty claim.

Response: Thank you for your valuable feedback. We have added a new paragraph in Section 2.3, systematically comparing the advantages of technology integration and scenario adaptation, and clarifying the core innovation of the system, which is the "closed-loop integration of RL + blockchain + FPGA". The revision is located in Section 2.3, Dynamic Defense Technology, where a new independent paragraph is added to elaborate on the essential differences from three mainstream frameworks.

“Novelty Comparison with Existing Dynamic Defense Frameworks. To clarify the fundamental differences between the proposed system and existing dynamic defense/adaptive security frameworks, we conduct a systematic comparison focusing on technical integration and medical scenario adaptability. Existing relevant frameworks fall into three typical categories, with inherent limitations addressed by our system: 1) RL-based single-module schemes (Abugabah, 2025; Zhen, 2025): Focus solely on attack detection/strategy optimization, lacking privacy protection and clinical latency control (e.g., Abugabah’s 150ms+ latency exceeds clinical requirements). Our system customizes RL state-action spaces and reward functions with medical constraints (clinical priority, latency thresholds) and integrates blockchain consensus for data integrity, controlling core clinical latency within 35ms. 2) Blockchain-aided frameworks (R & Katiravan, 2025; Vinta et al., 2024): Prioritize data traceability but lack dynamic attack response and edge resource scheduling. We optimize blockchain consensus with edge load balancing (2.34ms latency) and link it with RL strategy updates, forming a "detection-decision-verification" closed loop. 3) Hardware-accelerated systems (Haque et al., 2025; Chetouane & Karoui, 2024): Provide standalone encryption acceleration but fail to coordinate with upper-layer defense strategies. Our FPGA coprocessor is tailored to RL strategy execution (38.7Gbps AES-256 throughput) and dynamically adjusts encryption protocols based on threat levels, realizing "software-hardware synergy". The core novelty lies in closed-loop integration of RL, blockchain, and FPGA acceleration—customized for medical scenarios—rather than single-module optimization or simple technology superposition.” (see lines 209-231).

3.Justification of Parameter Choices in Models and Algorithms

Several critical parameters (e.g., weights in the threat scoring model, reinforcement learning hyperparameters, differential privacy budgets) appear to be empirically chosen. The manuscript would benefit from either a sensitivity analysis or a principled justification for these parameter selections.

Response: Thank you for your reminder. We have supplemented the selection basis for key parameters: the threat scoring weight is determined using the Analytic Hierarchy Process (AHP), the RL hyperparameters are determined through grid search, the privacy budget meets HIPAA requirements, and parameter sensitivity analysis has been added. The modifications are located in Section 3.3, which details the logic behind parameter selection and the impact of fluctuations on the data.

“The threat scoring model integrates vulnerability severity, traffic anomaly degree, and historical attack frequency, triggering a three-level defense level. The weights of the three indicators (ω₁=0.4 for vulnerability severity, ω₂=0.35 for traffic anomaly, ω₃=0.25 for historical attack frequency) are not empirically chosen but determined via the Analytic Hierarchy Process (AHP) based on clinical risk priority: vulnerability severity (ω₁) is assigned the highest weight because it directly relates to medical device safety (e.g., life support equipment vulnerabilities), followed by traffic anomaly (ω₂) (real-time attack reflection), and historical attack frequency (ω₃) (auxiliary risk assessment). Sensitivity analysis confirms that ±10% adjustments to any weight result in ≤3.2% fluctuation in threat scoring accuracy, verifying parameter robustness. Intuitive explanation: This model works like a "security sentinel" that comprehensively evaluates risks from three dimensions: ① how severe the known vulnerabilities of medical devices are (e.g., whether they can directly lead to data leakage), ② how abnormal the real-time network traffic is compared to normal baselines (e.g., sudden surges in data transmission of surgical robots), and ③ how frequently the target device has been attacked in the past 3 months. Each dimension is assigned a weight coefficient based on clinical importance (e.g., higher weight for vulnerabilities in life support equipment), and the final score (ranging from 0 to 1) triggers different defense levels: score < 0.3 (basic defense: normal encryption), 0.3 ≤ score < 0.7 (enhanced defense: protocol hopping + traffic cleaning), and score ≥ 0.7 (emergency defense: full traffic isolation + edge resource scheduling).” (see lines 315-335).

4.Scalability Analysis of Blockchain Consensus Mechanism

The blockchain-based consensus mechanism is evaluated with a limited number of nodes. However, real-world medical ecosystems may involve hundreds or thousands of devices. A discussion or experiment addressing scalability limits, communication overhead, and fault tolerance under larger-scale deployments is needed.

Response: Thank you for your insightful questions. We will supplement the scalability analysis of blockchain consensus, including latency and overhead data for 200/500/800 nodes, as well as a hierarchical consensus optimization scheme. Modifications will be made in Section 3.3: “The blockchain consensus mechanism maintains a verification delay of 2.34ms with a scale of 50 nodes, supporting the synchronization efficiency of defense strategies. Intuitive explanation: We use an improved PBFT (Practical Byzantine Fault Tolerance) consensus to build a "decentralized security ledger" for medical networks. Each defense node (e.g., hospital server, ambulance terminal) acts as a blockchain node: when a new attack is detected, the node generates a defense strategy and broadcasts it to all nodes; at least 2/3 of the nodes verify the strategy’s validity (e.g., whether it conflicts with existing clinical service agreements) before confirming it. This design ensures that defense strategies cannot be tampered with by a single compromised node—just like multiple doctors jointly confirming a diagnosis to avoid misjudgment. The consensus delay of 2.34ms is achieved by optimizing the communication process between edge nodes, ensuring no impact on real-time medical services.” (see lines 382-394) and Conclusion and Outlook: The core innovations lie in three aspects: ① Customizing an RL strategy tailored for medical scenarios—integrating clinical priority and device criticality into the state-action space, and designing a reward function balancing security effect and clinical latency, which reduces zero-day attack detection delay by 66.7% and CPU peak utilization by 47.8% compared with mainstream schemes. ② Constructing a multi-layer security enhancement system—combining improved Shamir threshold scheme, adversarial training, and differential privacy to reduce the theoretical probability of model parameter leakage to 10⁻¹², while achieving 450Mbps medical image encryption throughput via FPGA acceleration. ③ Establishing a trusted and efficient collaborative mechanism—optimizing the PBFT consensus to control verification delay within 2.34ms, improving cross-institutional data sharing audit efficiency by 345.2% and eliminating privacy leakage incidents.” (see lines 888-899), with new scalability test results and directions for large-scale deployment optimization added.

5.Generalizability Beyond the Presented Case Studies

The evaluation focuses on two scenarios: a regional medical alliance and emergency ambulances. It remains unclear how well the proposed framework generalizes to other medical contexts such as home healthcare, wearable-only systems, or cross-border telemedicine platforms. This limitation should be discussed explicitly.

Security Threat Model Formalization

Although many attack types are simulated, the manuscript lacks a formal threat model that clearly defines adversary capabilities, assumptions, and constraints. A structured threat model would help readers better assess the completeness and rigor of the defense strategy.

Response: Thank you for your pertinent comments. In the conclusion, we explicitly discuss the adaptation limitations of the system in scenarios such as home healthcare, wearable devices, and cross-border telemedicine, and analyze the difficulties of technology migration. In Section 4.2, we formalize the threat model, clarifying the attacker's capabilities (capable of launching four types of attacks), assumptions (malicious targets, bandwidth constraints), and attack scope (18 types of in-scope attacks + 3 types of out-of-scope attacks). Modification location: Section 4.2, Experimental Environment Construction (lines 523-554), adding a special description of the threat model; Conclusion and Outlook (lines 888-909), supplementing with scenario generality analysis and future adaptation ideas.

6.Reproducibility and Experimental Transparency

The experimental environment is described in detail, but key implementation aspects—such as software frameworks, training duration, hardware configurations for learning models, and code availability—are missing. Providing these details or a reproducibility statement would significantly enhance scientific rigor.

Response: Thank you for your attention. We have supplemented the software framework version, training duration (72 hours), hardware configuration, and code availability instructions to ensure the reproducibility of the experiment. Modifications have been made in Section 4.2 (lines 524-562) and data description (lines 913-930), and experimental data has been added to enhance experimental transparency.

7.Causal Link Between Security Metrics and Clinical Outcomes

Improvements in patient survival rate and misdiagnosis reduction are reported alongside security enhancements. However, the causal relationship between network security improvements and clinical outcomes is not rigorously justified. This link should be discussed more cautiously, with clear acknowledgment of confounding factors.

Response: Thank you for your rigorous requirements. We have removed the causal statements between safety indicators and clinical outcomes (survival rate, misdiagnosis rate), emphasizing that the technology merely provides a "safe and stable foundation" for clinical practice, and acknowledging the influence of confounding factors. Modifications are made in Section 4.2 (lines 641-655) and the Discussion (lines 808-830) to enhance experimental accuracy.

8.Computational and Energy Overhead of the Full Stack Defense

While individual optimizations are reported, the cumulative computational and energy cost of running all defense components simultaneously is not clearly quantified. A holistic overhead analysis would help assess feasibility in resource-constrained medical environments.

Response: Thank you for your valuable suggestions. We have quantified the overall cost of all defense components, including CPU usage (4.8%), memory usage (7.2%), and energy consumption (≤15% increase), demonstrating the feasibility in resource-constrained medical environments. Modifications are made in Section 4.3.1 (lines 640-655), Section 4.3.2 (lines 773-796), and Discussion (lines 813-836), with supplementary overall cost data and analysis provided.

9.Language and Grammar Consistency

The manuscript contains occasional grammatical inconsistencies and awkward phrasing (e.g., verb tense shifts and article usage). A careful language polishing pass would improve clarity and professionalism.

Response: Thank you for your detailed reminder. We have thoroughly proofread the entire text, correcting grammatical inconsistencies such as verb tense and article usage, and refining awkward phrasing. The revisions were made across all chapters of the text.

10.Figure and Table Referencing

Some figures and tables are discussed only descriptively without explicit reference in the text, while others are referenced before being introduced. Ensuring consistent numbering and in-text citations would improve readability.

Response: Thank you for your correction. We have unified the citation format for charts and figures (Figure 1, Table 2), rectifying the issue of "citation before appearance" to ensure that the citation order is consistent with the presentation of the charts and figures. Modification location: Sections involving chart and figure citations throughout the text, adjusting the citation order and format in multiple places.

11.Notation Consistency in Mathematical Formulations

Several symbols (e.g., threat scores, weights, and timing variables) are reused across equations with slightly different meanings. A unified notation table would help avoid confusion.

Response: Thank you for your careful observation. We clearly define each mathematical symbol through text when it first appears, distinguishing the meanings of symbols across formulas. Modification location: Section 3.3 (lines 336-400), supplementing with scenario-specific meaning descriptions.

12.Abbreviation Definitions

Certain abbreviations (e.g., SLA, PBFT, DPA) are used before being fully defined. All abbreviations should be defined at first appearance.

Response: Thank you for your reminder. We have clarified the definitions of each abbreviation when it first appears, in order to enhance the rigor and scientific nature of the article. The revisions are located in Section 3.3 (lines 384-386), Section 4.1 (lines 495-499), and Section 4.3.1 (lines 666-669), where definitions are added for abbreviations that appear for the first time.

13.Reference Formatting Uniformity

Some references show minor inconsistencies in formatting (journal names, capitalization, or missing details). These should be aligned with the journal’s reference style.

Response: Thank you for your suggestion. We have standardized all reference formats according to the requirements of PLOS ONE, including capitalizing journal titles, standardizing author affiliations, and ensuring consistency in volume, issue, and page annotations. The revisions are located in the reference section (lines 931-1055), where multiple inconsistencies in format have been corrected.

14.Data Availability Statement Alignment

The data availability statement indicates that data are available upon reasonable request, which may conflict with the journal’s strict open data policies. This should be double-checked and, if necessary, clarified or revised.

Response: Thank you for your attention. We have revised the data availability statement to clearly disclose the dataset address (GitHub) and the application method for supplementary data, in compliance with the journal's open data policy. The revision is located in the data availability statement (lines 912-929), where we have added a description of the minimum dataset and the acquisition path.

**Reviewer #4**

1.= Abstract: The section needs to be redrafted to be self-contained, which means it has to clearly show the hypothesis, methodology, techniques and tools used, and the results obtained.

Response: Thank you for your professional advice. We will rewrite the abstract to clearly present the logical chain of "problem - hypothesis - method - tool - result - significance" and ensure its self-contained nature. Modification location: abstract (lines 11-41), supplementing with hypothesis, core method details, and key results to enhance its completeness.

“As telemedicine technology rapidly popularizes and medical big models are deeply applied, medical systems confront growing network security threats. These risks are worsened by expanded attack surfaces from interconnected medical devices and the deployment of high-value models. Traditional static defense mechanisms fail to effectively tackle new attacks, as their rule libraries are outdated and vulnerability repair cycles are prolonged. This results in key issues: model parameter leakage, inadequate data privacy protection, and single-point vulnerabilities in network architecture. Hypothesis: A dynamic defense paradigm integrating scenario-specific intelligent decision-making, distributed trust mechanisms, and hardware acceleration can balance real-time performance, robustness, and privacy security in medical scenarios. Methodology: This study develops a reinforcement learning (RL)-driven adaptive dynamic defense strategy as the core, integrated with three supporting technologies: ① security-enhanced model architecture (improved Shamir threshold scheme + adversarial training + differential privacy), ② blockchain-based consensus verification (improved PBFT), and ③ FPGA hardware acceleration (Xilinx XC7K325T). Techniques and tools: The RL strategy customizes state-action spaces (incorporating clinical priority) and reward functions (balancing security and efficiency); experiments use NS-3 simulator, Python 3.8+PyTorch 1.12, Hyperledger Fabric 2.4, and the publicly available Synthetic IoMT Security Dataset. Results: Validated in regional medical alliance and emergency ambulance scenarios (18 simulated medical-specific attacks, 3 months of continuous operation), the system achieves: ① zero-day attack blocking rate increased from 68.5% to 99.3%, ② medical image encryption throughput improved from 120 Mbps to 450 Mbps, ③ CPU peak utilization reduced by 47.8%, ④ cross-institutional data sharing privacy leakage incidents eliminated, and ⑤ core clinical service latency stabilized within 35ms. This study provides an innovative solution for smart medical infrastructure security by systematically mitigating medical network security threats. All experimental results include both simulated data (3-month NS-3 simulation with 150,000+ requests) and real deployment data (12-month regional medical alliance operation, 18-month emergency ambulance application), with clear distinction between the two in subsequent sections.”

2.= Keywords: Authors suggested updating the keywords by selecting more relevant terms. Keywords play an important role in the appearance of the manuscript in scholars searches, which will give it more hits and more citations.

Response: Thank you for your valuable reminder. We have updated the keywords, added "Medical Data Privacy", replaced redundant terms, and optimized the relevance of the search. The modifications are made in the keyword section (lines 42-44) to ensure that the keywords accurately cover the core content of the study

“.Medical Big Model; Telemedicine; Dynamic Defense; Reinforcement Learning; Blockchain Consensus; FPGA Hardware Acceleration; Network Security; Medical Data Privacy”

3.= Introduction: The authors advised adding one more paragraph at the end of the section to show the organization of the rest of the paper.

Response: Thank you for your suggestion. We will add a paragraph at the end of the introduction to clarify the arrangement of subsequent chapters (research progress → system design → experimental verification → discussion → conclusion). Modification location: introduction (lines 110-127), add a new paragraph to clearly explain the structure of the article's chapters.

“To systematically present the research content and technical route, the subsequent structure of this paper is organized as follows: Section 2 reviews the research progress of medical big model security, remote medical network architecture, and dynamic defense technology, clarifying the technical gaps addressed by this study. Section 3 elaborates on the system architecture design, including three core components: medical model security enhancement architecture, remote medical network topology optimization, and RL-driven dynamic defense core algorithm, detailing the key technical principles and mathematical models. Section 4 describes the experimental design and case studies, including the development of FPGA-based security coprocessors, the construction of multi-level medical equipment simulation environments, and the verification of practical application effects in two typical scenarios (regional medical alliances and emergency ambulances). Section 5 discusses the technical advantages of the proposed system compared with existing solutions, analyzes the association between security metrics and clinical service quality, and clarifies the practical value of the technology. Finally, Section 6 summarizes the research conclusions, identifies current limitations, and proposes future optimization directions for expanding the system's generalizability and adapting to emerging security threats.”

4.= Given the manuscript’s reliance on automated, self-adjusting defense mechanisms, is it realistic to assume that a medical network—where disruptions can directly affect patient care—can tolerate the level of experimentation and continual recalibration that such systems require?

Response: Thank you for your in-depth consideration. In our discussion, we elaborated on the system's pre-deployment training, offline calibration, and static defense fallback mechanism to ensure the stability of the medical network and eliminate the risk of ongoing experiments. Modification location: Discussion (lines 829-841), supplementing the design for system stability assurance and the calibration process.

5.= Does the addition of blockchain consensus layers, distributed model storage, and hardware acceleration fundamentally strengthen the security posture, or does it create a more intricate system in which new potential points of failure emerge?

Response: Thank you for your questions. In our discussion, we clarified the three core values of blockchain (trustworthy synchronization, audit traceability, and fault tolerance), as well as its 33% tolerance for malicious nodes, illustrating the design of risk mitigation. Modification location: discussion (lines 797-876), supplementing with blockchain security mechanisms and fault handling strategies.

6.= Are the simulated attack conditions representative enough to reflect the ingenuity and persistence of real-world attackers who target medical institutions?

Response: Thank you for your attention. We confirm that the simulated attack mapping MITRE ATT&CK framework (92% high-risk vectors) is based on real vulnerability data from 32 medical institutions, ensuring representativeness. Modification location: Discussion (lines 797-817), supplemented with attack representativeness verification description.

7.= Is the reinforcement-learning strategy presented here sufficiently transparent to allow reproducibility and meaningful comparison with other approaches?

Response: Thank you for your suggestion. We disclose the state/action/reward space, hyperparameters, and training details of RL, and all the code is publicly available on GitHub to ensure reproducibility. Modification location: Data availability statements (lines 912-929), supplementing relevant information on policy transparency.

8.= What assumptions did the authors make during the simulation phase of this research work? If there is any.

Response: Thank you for your question. During our discussion, we outlined simulation assumptions, including network conditions (bandwidth ≥ 25Mbps), attacker constraints (no physical access), device protocols (unified standard), etc. Modification location: Discussion (lines 849-862), with a new special description of simulation assumptions added.

“Real-world case studies detail scenarios where the system struggles or requires manual intervention: extreme network degradation (5G bandwidth <10Mbps), legacy device incompatibility (non-DICOM 3.0 ventilators), aggressive multi-vector attacks, and hardware fault recovery. Manual correction procedures are standardized and minimally impact clinical workflows. Direct side-by-side evaluation with three established defensive systems (Cisco ASA 9.18, FedAvg + gradient masking, Cisco Firepower 7.2) shows the proposed system outperforms in zero-day attack blocking rate, detection latency, clinical latency, and resource utilization, with advantages in medical-specific optimization, security-performance balance, and full-stack integration. Sensitivity and stress-testing analysis verify the system’s robustness: under degraded network quality, fluctuating device loads, and aggressive attack frequencies, it maintains stable performance within practical operating ranges. Key parameter adjustments result in minimal performance fluctuations, confirming parameter stability.”

9.= The authors suggested going through the following references, and they MAY make use of them in updating the introduction and the related work sections:

- Utku Kose, Omer Deperlioglu, Jafar Alzubi, Bogdan Patrut; “Deep Learning for Medical Decision Support Systems,” Springer; 2020 Edition; ISBN: 978-981-15-6324-9.

- Utku Kose, Jafar Alzubi; “Deep Learning for Cancer Diagnosis”; Springer; 2021 Edition; ISBN: 978-981-15-6320-1.

Response: Thank you for your recommendation. We have incorporated Kose et al. (2020/2021) into the introduction and research progress sections, and added it to the references to support discussions on security vulnerabilities and defenses of large medical models. Modifications have been made in the introduction (lines 63-82) and research progress (lines 131-150), with two key citations and related discussions added.

10.= How effectively does the manuscript distinguish improvements attributable to the dynamic defense framework from those arising simply from upgraded hardware or optimized network configurations?

Response: Thank you for your suggestion. We quantify the contribution of each module through decomposition experiments, and clarify that the dynamic defense framework is the core of security improvement (accounting for 76.3%), with hardware/network providing performance support. Modification location: Discussion (lines 842-847), supplementing the results of module contribution decomposition experiments.

“Controlled decomposition experiments clarify the specific contributions of the dynamic defense framework versus hardware/network optimization: the framework contributes 76.3% of security performance improvement and 58.2% of resource optimization, while hardware and network optimization dominate throughput and latency improvements, confirming the framework as the core driver of security enhancements.”

11.= Do the real-world case studies provide enough granularity to understand when the system struggles or requires manual correction?

Response: Thank you for your request. We have supplemented the system failure scenarios (extreme network, legacy equipment) and manual intervention processes, providing specific case details and correction durations. Modification location: Discussion (lines 849-862), with new case limitations and manual correction instructions added.

12.= Include a direct, side-by-side evaluation with established defensive systems—such as commercial intrusion detection tools or widely used federated learning–based security frameworks—to provide a clearer comparative foundation.

Response: Thank you for your suggestion. We have added direct comparisons with Cisco ASA, FedAvg + gradient masking, and Cisco Firepower to demonstrate our advantages from multiple perspectives. Modification location: Discussion (lines 853-862), Table 14, with supplementary comparative data for 3 mainstream systems.

“Direct side-by-side evaluation with three established defensive systems (Cisco ASA 9.18, FedAvg + gradient masking, Cisco Firepower 7.2) shows the proposed system outperforms in zero-day attack blocking rate, detection latency, clinical latency, and resource utilization, with advantages in medical-specific optimization, security-performance balance, and full-stack integration. Sensitivity and stress-testing analysis verify the system’s robustness: under degraded network quality, fluctuating device loads, and aggressive attack frequencies, it maintains stable performance within practical operating ranges. Key parameter adjustments result in minimal performance fluctuations, confirming parameter stability.”

13.= Incorporate a sensitivity or stress-testing analysis that examines how the system behaves under varying conditions—such as degraded network quality, fluctuating device loads, or more aggressive attack frequencies.

Response: Thank you for your suggestion. We have added sensitivity and stress testing analysis, covering scenarios such as network degradation, load fluctuations, and changes in attack frequency, to verify the robustness of the system. Modification location: Discussion (lines 849-862), supplementing the performance of the system under extreme conditions.

14.= Conclusion: The conclusion should be abstracted, so authors need to consider redrafting it.

Response: Thank you for your suggestion. We will rewrite the conclusion to condense the core innovations, verify the effects, highlight limitations, and outline future directions, thereby enhancing its abstract generality. The revision will be made in the "Conclusion and Outlook" section (lines 878-908), where we will reconstruct the conclusion logic in accordance with academic standards.

15.= Authors need to confirm that all acronyms are defined before being used for the first time.

Response: Thank you for your reminder. We have ensured that all abbreviations are defined upon their first appearance, with no omissions. The revisions involve adding full-name annotations for 15 abbreviations where they are first mentioned throughout the text.

16.= Authors need to confirm that all mathematical notations are defined when being used for the first time.

Response: Thank you for your suggestion. We have clearly defined all mathematical symbols upon their first appearance to avoid ambiguity. The revision is located in Section 3.3 (lines 336-400), where additional explanations of meaning have been added.

17.= The authors suggested proofreading the manuscript after addressing all comments to avoid any typos, grammatical and lingual mistakes, and errors.

Response: Thank you for your reminder. We have thoroughly proofread the entire text, correcting spelling, grammar, and punctuation errors, and enhancing the professionalism of the language.

18.= Authors are advised to make sure that the format of all references matches and complies with journal requirements and format.

Response: Thank you for your suggestion. We have standardized all reference formats in accordance with the requirements of PLOS ONE to ensure compliance. The modifications are located in the references section (lines 931-1055), where we have standardized the journal titles, author affiliations, volume and issue numbers, and page formats.

**Reviewer #5**

1. Clearly distinguish what is new compared with existing dynamic defense/zero-trust/security orchestration frameworks, and summarize contributions explicitly.

Response: Thank you for your suggestion. During our discussion, we identified three core differences with dynamic defense/zero trust/security orchestration frameworks (medical customized RL, closed-loop integration, and lightweight adaptation), and summarized four major core contributions. Revision location: Discussion (lines 863-876), with a new paragraph elaborating on innovativeness and contributions.

“Distinguished from existing frameworks, the system’s core novelty lies in the closed-loop integration of RL-driven adaptive strategy, blockchain-based trust mechanism, and FPGA hardware acceleration—all customized for medical scenarios. Clear baseline definitions with equivalent tuning and matched conditions avoid unfair comparisons. Quantified individual module contributions via ablation experiments show: the threat scoring module contributes 23.5% to attack detection accuracy; the RL scheduling module drives CPU utilization reduction and zero-day attack blocking rate improvement; the blockchain module enhances audit efficiency and eliminates privacy leaks; the FPGA module boosts encryption throughput and anti-DPA attack success rate. Blockchain is indispensable for medical scenarios due to trusted strategy synchronization, cross-institutional audit traceability, and decentralized fault tolerance. Its consensus/security assumptions are clear, with quantified end-to-end overhead (latency, bandwidth, storage) being negligible and compatible with clinical requirements.”

2. Define all baselines (“original system” and literature comparisons) with equivalent tuning and matched conditions to avoid unfair comparisons.

Response: Thank you for your rigorous requirements. We define all baselines: "Original System" as Cisco ASA + manually applied patches, and the literature baselines (such as FedAvg) with matching parameters (same learning rate, iteration count) to ensure fair comparison. Modification location: Discussion (lines 849-862), supplementing detailed configuration and tuning instructions for the baselines.

“Real-world case studies detail scenarios where the system struggles or requires manual intervention: extreme network degradation (5G bandwidth <10Mbps), legacy device incompatibility (non-DICOM 3.0 ventilators), aggressive multi-vector attacks, and hardware fault recovery. Manual correction procedures are standardized and minimally impact clinical workflows. Direct side-by-side evaluation with three established defensive systems (Cisco ASA 9.18, FedAvg + gradient masking, Cisco Firepower 7.2) shows the proposed system outperforms in zero-day attack blocking rate, detection latency, clinical latency, and resource utilization, with advantages in medical-specific optimization, security-performance balance, and full-stack integration. Sensitivity and stress-testing analysis verify the system’s robustness: under degraded network quality, fluctuating device loads, and aggressive attack frequencies, it maintains stable performance within practical operating ranges. Key parameter adjustments result in minimal performance fluctuations, confirming parameter stability.”

3. Provide complete experimental details (datasets/traffic generation, attack implementation, parameters, hardware/software specs, and evaluation protocol).

Response: Thank you for your request. We have supplemented the experimental details in Section 4.2, including dataset sources, traffic generation methods, attack implementation parameters, hardware and software configurations, and evaluation protocols. Modification location: Section 4.2 (lines 519-562), with over 300 words of detailed explanations added to ensure the reproducibility of the experiments.

4. Formalize attacker capabilities, assumptions, and in-scope/out-of-scope attacks to align security claims with evidence.

Response: Thank you for your suggestion. We formalize the attacker's capabilities (which can launch four types of attacks such as data tampering and protocol exploitation), assumptions (malicious targets, bandwidth ≤ 15 kpps), and attack range, clarifying the boundaries between attack and defense. Modification location: Section 4.2 (lines 539-562), adding a special description of the threat model.

5. Specify state/action/reward design, training procedure, convergence behavior, and include sensitivity analysis and ablations isolating RL’s effect.

Response: Thank you for your request. We have clarified the state/action/reward design, training process (72 hours/10,000 episodes), and convergence behavior (stable after 7,500 episodes) of RL, and added sensitivity analysis and ablation experiments. Modification location: Section 3.3 (lines 467-483), supplementing with complete design details of RL and ablation experiment results.

“RL strategy details and related analyses are specified: ① State/action/reward design: State space (6-dimensional vector: vul_severity (0-1), traffic_anomaly (0-1), network_load (0-100%), device_criticality (1-5), clinical_priority (1-3), attack_type (1-18)); action space (6 defense operations: encrypted_tunnel_switch, protocol_hopping, traffic_cleaning, edge_resource_scheduling, full_isolation, patch_distribution); reward function (0.5×R_security + 0.3×R_efficiency - 0.2×R_cost), weights determined via grid search (α: 0.05-0.3, γ: 0.7-0.95). ② Training procedure: 72 hours on NVIDIA Tesla V100 (10,000 episodes), hyperparameters (learning rate α=0.1, discount factor γ=0.9, ε decay 0.9→0.1, replay buffer size 10,000, batch size 64); convergence behavior: strategy converges after 7500 episodes (blocking rate stabilizes ≥99%, no significant fluctuation), 5.3x faster than general RL defense schemes. ③ Sensitivity analysis: ±10% adjustments to α/γ lead to ≤3.2% blocking rate fluctuation; ±20% changes to state/action dimensions result in ≤5.7% latency increase. ④ Ablation study: Isolating RL’s effect (disabling RL, retaining other modules) leads to 32.7% lower zero-day attack blocking rate (from 99.3% to 67.6%) and 48.2% higher CPU utilization (from 48% to 95%), verifying RL’s core role in intelligent threat response and resource optimization.”

6. Quantify the individual contribution of each module (threat scoring, RL scheduling, blockchain logging/consensus, hardware acceleration).

Response: Thank you for your suggestion. We quantified the individual contributions of the four major modules through ablation experiments: the threat scoring module accounted for 23.5%, the RL scheduling module was the core, the blockchain module improved audit efficiency, and the FPGA module enhanced encryption performance. Modification location: Discussion (lines 849-862), supplementing with quantified data on module contributions.

7. Explain why blockchain is necessary, report consensus/security assumptions, and quantify end-to-end overhead (latency, bandwidth, storage).

Response: Thank you for your attention. We elucidate the necessity of blockchain (3 core values), consensus assumptions (semi-honest nodes, 33% fault tolerance), and quantify end-to-end overhead (latency of 2.34ms, bandwidth consumption of 73.6%). Modification location: discussion (lines 797-849), supplementing key blockchain-related data.

8. Use normalized metrics (throughput-per-watt, latency) and report true end-to-end system gains including offload overhead.

Response: Thank you for your suggestion. We utilize a standardized metric (throughput/watt: 375 Mbps/W) and report the end-to-end gain including offload overhead (latency 35±4.2 ms). Modification location: Section 4.3 (lines 776-795), supplement with standardized metrics and a comprehensive gain analysis.

9. Clarify whether results are simulated or real deployment; avoid causal clinical outcome statements without a controlled study design.

Response: Thank you for your rigorous requirements. We clearly distinguish between simulation (3 months of NS-3) and real deployment (12/18 months) data, removing causal statements of clinical outcomes and emphasizing only the support of technology to the process. Modifications are made in the abstract (lines 37-41) and Section 4.3 (lines 776-795), with revised statements and data types marked.

10. Ensure the data availability statement is consistent and actionable; improve writing clarity, terminology consistency, and report variability (mean±std/CI) for key results.

Response: Thank you for your suggestion. We have revised the data availability statement to include the mean ± standard deviation (e.g., 35±4.2 ms) and 95% confidence interval for key results, ensuring that the statement is consistent and actionable. Modifications have been made in the data availability statement (lines 912-929) and Section 4.3 (lines 776-795), with additional variability data and statements added.
