# Supplementary material for: Remote medical system driven by medical big models: Dynamic defense model for network security threats
Source: PLoS One. 2026 Jun 12;21(6):e0348572. doi: 10.1371/journal.pone.0348572 (PMC13262880; doi:10.1371/journal.pone.0348572)
Supplement: Attachment — Submitted filename: Response_to_Reviewers_auresp_2.docx [file pone.0348572.s005.docx]

**Dear Editor and Reviewer**:

Thank you very much for your valuable feedback on our manuscript. We have carefully studied each comment from this round of review and made corresponding modifications and verifications to the manuscript based on the actual content of this revised version.

Journal Requirements:

1. If the reviewer comments include a recommendation to cite specific previously published works, please review and evaluate these publications to determine whether they are relevant and should be cited. There is no requirement to cite these works unless the editor has indicated otherwise.

Response: Thank you for the reminder from the editorial department. We have rechecked the citations in the full text based on the review feedback. There was no requirement in this round of review to add specific literature, so we did not mechanically include references that are not sufficiently relevant to the topic of this article. Instead, we carefully retained and systematically organized the references based on their relevance to the research topic, methodological support, and necessity for the argumentation of this article.

2. Please review your reference list to ensure that it is complete and correct. If you have cited papers that have been retracted, please include the rationale for doing so in the manuscript text, or remove these references and replace them with relevant current references. Any changes to the reference list should be mentioned in the rebuttal letter that accompanies your revised manuscript. If you need to cite a retracted article, indicate the article’s retracted status in the References list and also include a citation and full reference for the retraction notice.

Response: We have rechecked the reference list according to this requirement and unified the presentation format of references in this revision, including author writing style, title case, journal name presentation, and volume (issue) and page/article number writing style. At the same time, we also rechecked the completeness and consistency of the reference list and truthfully described the revisions related to references in this reply letter.

Reviewer #1: The manuscript addresses an important and timely problem in telemedicine network security and proposes a reinforcement learning driven dynamic defense framework. The work is technically sound, well structured, and significantly improved following revision. The integration of RL, blockchain, and hardware acceleration is clearly presented, and experimental validation is reasonably detailed.

(1) However, minor issues remain. The manuscript would benefit from tighter focus on the core contribution, clearer interpretation of performance claims, and minor language polishing.

Response: Thank you for the further suggestions from the reviewer. Based on this feedback, we have continued to strengthen the centralized expression of core contributions in this revised manuscript, especially in the Abstract, Introduction, and Discussions sections. The core contributions of this paper are clearly focused on: an RL-driven adaptive dynamic defense strategy for telemedicine scenarios, and its closed-loop integration with model protection architecture, blockchain-based verification, and FPGA acceleration. At the same time, we have adopted more cautious academic wording for performance descriptions, such as using expressions like "under the evaluated conditions," "suggest," and "indicate" more explicitly in the abstract, case analysis, and discussion sections. When discussing business support capabilities, we emphasize their technical significance and avoid directly extrapolating technical results to clinical outcome improvements. We have also further refined some sentences in the context of the full text to enhance the accuracy and coherence of expression.

(2) Some sections are dense and could be simplified for readability.

Response: Thank you for the pertinent suggestions from the reviewer. We have further compressed and reorganized some information-dense paragraphs based on this feedback, focusing on reducing repetitive narration of table values and enhancing the generality and explanatory nature of the paragraphs. Especially in 4.3 Case Analysis, we have rewritten the interpretation of the results after case analysis: in 4.3.1, we compressed the descriptions following Tables 7–9, and in 4.3.2, we rewrote the analysis paragraphs corresponding to Table 10, Figure 10, Figure 11, Table 11, and Table 12. While retaining the references to tables and figures, we changed the order to first summarize the main findings and then explain their significance in the emergency ambulance scenario, thereby improving readability. For 3.3 Dynamic Defense Core Algorithm, we retained the necessary formulas and parameter descriptions to support the integrity of the method, while minimizing repetitive results-oriented expressions, aiming to strike a balance between technical completeness and reading fluency.

(3) Clarification on generalizability and practical deployment limitations would further strengthen the work.

Response: Thank you for the reviewer's suggestions. We have further strengthened the explanation of generalization and practical deployment limitations in this revised manuscript. Specifically, we emphasize in Section 5 Discussions that the improvements reported in this paper primarily manifest as technical support for the security, resilience, and operational continuity of telemedicine systems, and should not be directly interpreted as clinical outcome improvements. Additionally, we have set up a separate Section 6, "Limitations and Generalizability," to provide a more systematic explanation of the scope of application and deployment boundaries, including: generalization limitations across different telemedicine infrastructures, verification level differences between simulation results and deployment observations, dependence on FPGA and edge computing capabilities, potential constraints when scaling blockchain node sizes, and issues such as physical tampering, quantum cryptanalysis, and nation-state-level attacks that are not currently covered by the threat model. We hope these additions can present the applicable boundaries of this paper more authentically and cautiously.

“At the same time, the reported improvements should be interpreted within the scope of the evaluated scenarios(Table 13). The proposed framework is intended to provide a secure and stable technical support layer for remote medical services rather than to establish direct causal effects on clinical outcomes. In this sense, improvements in attack blocking, transmission stability, and workflow continuity may support medical operations, but they should not be interpreted as direct evidence of improved diagnosis accuracy, reduced misdiagnosis, or improved patient survival. The practical value of the framework therefore lies in strengthening the security, resilience, and operational continuity of remote medical systems.” (see lines 549-557).

(4) Ethical and data transparency aspects are generally appropriate, but authors should confirm originality and ensure full compliance with data availability requirements.

Response: Thank you for the reviewer's recognition and reminder. Regarding research ethics and data transparency, we have further retained and verified the Ethical approval and informed consent statements as well as the Data availability statements at the end of the revised manuscript, clearly stating the ethical approval information for the supplementary de-identified clinical data (Approval No. SZPH-2023-089), the method of data de-identification processing, the source of the public dataset, and the conditions for obtaining data that cannot be publicly shared due to privacy and institutional restrictions. Regarding originality confirmation, we hereby solemnly state that this manuscript is the original research work of the author team. In this revision, we have also rechecked the relevant expressions to ensure that the data sources, ethical information, and availability descriptions are consistent, complete, and transparent with the content of the manuscript.

Reviewer #4: The authors have carefully revised and improved the manuscript based on reviewer feedback ‎from the previous review cycle. As a result, the manuscript now meets the journal's standards. ‎(1) However, a detailed review reveals some linguistic and grammatical issues throughout. To ‎address these, the authors are strongly encouraged to have the manuscript proofread by a ‎native English speaker. This will help fix any remaining language or grammatical errors and ‎ensure the manuscript reads smoothly and clearly.

Response: Thank you for the further suggestions from the reviewer. We have carefully understood the issues pointed out in this comment and continued to conduct a paragraph-by-paragraph examination and language polishing of the entire text in this revision. We focused on further unifying the expressions in the abstract, introduction, case analysis, discussion, and conclusion sections to improve the cohesion between sentences, consistency of terminology, and the robustness of academic expression. For sentences involving result interpretation, we paid special attention to avoiding overly strong inferences to keep them consistent with the evidence hierarchy of this article. We will continue to take language quality issues seriously and make our utmost efforts to further enhance the overall readability and clarity of expression of the manuscript.

(2) Furthermore, the authors must carefully ‎check and confirm that all references comply with the journal's required style and format. ‎Maintaining consistency and accuracy in referencing is vital for upholding the manuscript's ‎professionalism and integrity. By addressing these language and citation concerns, the ‎authors can significantly improve the clarity, readability, and overall quality of their work, ‎thereby supporting a smoother publication process and better dissemination of their ‎research.‎

Response: Thank you for the reminder from the reviewer. We have rechecked and unified the reference format in this revision, and standardized the reference list as a whole. The main changes include: unifying the author presentation method, standardizing the capitalization of titles, regulating the writing of journal names, and unifying the presentation of volume (issue) and page/article numbers. We have also rechecked the completeness and consistency of the reference list to reduce inconsistencies in format. We strongly agree with the reviewer's opinion on the importance of reference normalization to the professionalism and readability of the manuscript, and have accordingly conducted a centralized organization of the References section at the end of the manuscript.

We would like to extend our gratitude to the editors and reviewers for their meticulous review and constructive feedback on our manuscript. We sincerely hope that this revision and our detailed responses can address everyone's concerns and further enhance the quality of our manuscript.

Sincerely,

The Authors
