# Supplementary material for: Remote medical system driven by medical big models: Dynamic defense model for network security threats
Source: PLoS One. 2026 Jun 12;21(6):e0348572. doi: 10.1371/journal.pone.0348572 (PMC13262880; doi:10.1371/journal.pone.0348572)
Supplement: Peer review history

# Peer review: Decision Letter 0

**DOI:** 10.1371/journal.pone.0348572.r001

**Authors:** Raman Singh

18 Jan 2026

PONE-D-25-50491

Remote Medical System Driven by Medical Big Models: Dynamic Defense Model for Network Security Threats

PLOS One

Dear Dr. Hu,

Thank you for submitting your manuscript to PLOS ONE. After careful consideration, we feel that it has merit but does not fully meet PLOS ONE’s publication criteria as it currently stands. Therefore, we invite you to submit a revised version of the manuscript that addresses the points raised during the review process.

Please submit your revised manuscript by Mar 04 2026 11:59PM. If you will need more time than this to complete your revisions, please reply to this message or contact the journal office at plosone@plos.org. When you're ready to submit your revision, log on to https://www.editorialmanager.com/pone/ and select the 'Submissions Needing Revision' folder to locate your manuscript file.

Please include the following items when submitting your revised manuscript:

- A letter that responds to each point raised by the academic editor and reviewer(s). You should upload this letter as a separate file labeled 'Response to Reviewers'.
- A marked-up copy of your manuscript that highlights changes made to the original version. You should upload this as a separate file labeled 'Revised Manuscript with Track Changes'.
- An unmarked version of your revised paper without tracked changes. You should upload this as a separate file labeled 'Manuscript'.

If you would like to make changes to your financial disclosure, please include your updated statement in your cover letter. Guidelines for resubmitting your figure files are available below the reviewer comments at the end of this letter.

If applicable, we recommend that you deposit your laboratory protocols in protocols.io to enhance the reproducibility of your results. Protocols.io assigns your protocol its own identifier (DOI) so that it can be cited independently in the future. For instructions see: https://journals.plos.org/plosone/s/submission-guidelines#loc-laboratory-protocols. Additionally, PLOS ONE offers an option for publishing peer-reviewed Lab Protocol articles, which describe protocols hosted on protocols.io. Read more information on sharing protocols at https://plos.org/protocols?utm_medium=editorial-email&utm_source=authorletters&utm_campaign=protocols.

We look forward to receiving your revised manuscript.

Kind regards,

Raman Singh

Academic Editor

PLOS One

Journal Requirements:

When submitting your revision, we need you to address these additional requirements.

1.Please ensure that your manuscript meets PLOS ONE's style requirements, including those for file naming. The PLOS ONE style templates can be found at

https://journals.plos.org/plosone/s/file?id=wjVg/PLOSOne_formatting_sample_main_body.pdf and

https://journals.plos.org/plosone/s/file?id=ba62/PLOSOne_formatting_sample_title_authors_affiliations.pdf

2. PLOS requires an ORCID iD for the corresponding author in Editorial Manager on papers submitted after December 6th, 2016. Please ensure that you have an ORCID iD and that it is validated in Editorial Manager. To do this, go to ‘Update my Information’ (in the upper left-hand corner of the main menu), and click on the Fetch/Validate link next to the ORCID field. This will take you to the ORCID site and allow you to create a new iD or authenticate a pre-existing iD in Editorial Manager.

3. Please note that PLOS One has specific guidelines on code sharing for submissions in which author-generated code underpins the findings in the manuscript. In these cases, we expect all author-generated code to be made available without restrictions upon publication of the work. Please review our guidelines at https://journals.plos.org/plosone/s/materials-and-software-sharing#loc-sharing-code and ensure that your code is shared in a way that follows best practice and facilitates reproducibility and reuse.

4. We note that the grant information you provided in the ‘Funding Information’ and ‘Financial Disclosure’ sections do not match.

When you resubmit, please ensure that you provide the correct grant numbers for the awards you received for your study in the ‘Funding Information’ section.

5. Please note that funding information should not appear in any section or other areas of your manuscript. We will only publish funding information present in the Funding Statement section of the online submission form. Please remove any funding-related text from the manuscript.

6. We note that your Data Availability Statement is currently as follows: “All relevant data are within the manuscript and its Supporting Information files.”

Please confirm at this time whether or not your submission contains all raw data required to replicate the results of your study. Authors must share the “minimal data set” for their submission. PLOS defines the minimal data set to consist of the data required to replicate all study findings reported in the article, as well as related metadata and methods (https://journals.plos.org/plosone/s/data-availability#loc-minimal-data-set-definition).

For example, authors should submit the following data:

- The values behind the means, standard deviations and other measures reported;

- The values used to build graphs;

- The points extracted from images for analysis.

Authors do not need to submit their entire data set if only a portion of the data was used in the reported study.

If your submission does not contain these data, please either upload them as Supporting Information files or deposit them to a stable, public repository and provide us with the relevant URLs, DOIs, or accession numbers. For a list of recommended repositories, please see https://journals.plos.org/plosone/s/recommended-repositories.

If there are ethical or legal restrictions on sharing a de-identified data set, please explain them in detail (e.g., data contain potentially sensitive information, data are owned by a third-party organization, etc.) and who has imposed them (e.g., an ethics committee). Please also provide contact information for a data access committee, ethics committee, or other institutional body to which data requests may be sent. If data are owned by a third party, please indicate how others may request data access.

7. If the reviewer comments include a recommendation to cite specific previously published works, please review and evaluate these publications to determine whether they are relevant and should be cited. There is no requirement to cite these works unless the editor has indicated otherwise.

Additional Editor Comments:

The manuscript require more work before it can be considered further. Please note that if a reviewer has advised to consider/cite some research papers, authors should use their own analysis if these suggested articles are actually required or not.

[Note: HTML markup is below. Please do not edit.]

Reviewer's Responses to Questions

**Comments to the Author**

1. Is the manuscript technically sound, and do the data support the conclusions?

The manuscript must describe a technically sound piece of scientific research with data that supports the conclusions. Experiments must have been conducted rigorously, with appropriate controls, replication, and sample sizes. The conclusions must be drawn appropriately based on the data presented.

Reviewer #1: Yes

Reviewer #2: No

Reviewer #3: Yes

Reviewer #4: Yes

Reviewer #5: Yes

**********

2. Has the statistical analysis been performed appropriately and rigorously?

Reviewer #1: Yes

Reviewer #2: No

Reviewer #3: N/A

Reviewer #4: Yes

Reviewer #5: Yes

**********

3. Have the authors made all data underlying the findings in their manuscript fully available?

The PLOS Data policy requires authors to make all data underlying the findings described in their manuscript fully available without restriction, with rare exception (please refer to the Data Availability Statement in the manuscript PDF file). The data should be provided as part of the manuscript or its supporting information, or deposited to a public repository. For example, in addition to summary statistics, the data points behind means, medians and variance measures should be available. If there are restrictions on publicly sharing data—e.g. participant privacy or use of data from a third party—those must be specified.

Reviewer #1: Yes

Reviewer #2: No

Reviewer #3: No

Reviewer #4: Yes

Reviewer #5: Yes

**********

4. Is the manuscript presented in an intelligible fashion and written in standard English?

PLOS ONE does not copyedit accepted manuscripts, so the language in submitted articles must be clear, correct, and unambiguous. Any typographical or grammatical errors should be corrected at revision, so please note any specific errors here.

Reviewer #1: Yes

Reviewer #2: No

Reviewer #3: No

Reviewer #4: Yes

Reviewer #5: Yes

**********

5. Review Comments to the Author

Please use the space provided to explain your answers to the questions above. You may also include additional comments for the author, including concerns about dual publication, research ethics, or publication ethics. (Please upload your review as an attachment if it exceeds 20,000 characters)

Reviewer #1: The manuscript presents a comprehensive and technically detailed study on a dynamic defense framework for remote medical systems driven by large medical models. The topic is timely and highly relevant given the increasing reliance on telemedicine and the growing security threats faced by interconnected medical infrastructures. Overall, the work demonstrates strong technical depth, clear motivation, and significant practical relevance, and it makes a valuable contribution to the fields of medical network security and intelligent protection systems.

The proposed multi-level dynamic defense architecture is well-structured and integrates advanced techniques such as reinforcement learning–based strategy optimization, blockchain consensus mechanisms, adaptive network protection, and hardware acceleration. The system design is coherent, and the closed-loop coordination between threat detection, defense decision-making, and resource scheduling is convincingly presented. The experimental evaluation is extensive, covering both simulated environments and real-world case studies, and the reported performance improvements (e.g., attack blocking rate, latency reduction, resource utilization, and system robustness) are impressive and well supported by quantitative results.

The manuscript is generally well organized, and the figures, tables, and case analyses help illustrate the effectiveness and applicability of the proposed approach in realistic medical scenarios, including regional medical alliances and emergency ambulance systems. The discussion and conclusion appropriately summarize the contributions and outline meaningful directions for future work.

That said, a few minor revisions would further strengthen the manuscript:

1. Clarity and language: Some sections would benefit from minor English-language polishing to improve readability and fluency. In particular, simplifying long sentences and ensuring consistent terminology (e.g., “medical big model,” “large medical model”) would enhance clarity.

2. Methodological clarification: While the technical framework is rich, brief intuitive explanations (in addition to mathematical formulations) for key components such as the threat scoring model, reinforcement learning strategy updates, and blockchain consensus integration would help readers from a broader audience better understand the design choices.

3. Reproducibility and comparison: The experimental section is thorough, but adding a short discussion on reproducibility (e.g., parameter sensitivity, computational overhead, or deployment constraints) and clarifying how baseline methods were selected would further improve transparency.

4. Limitations: Including a short paragraph explicitly discussing limitations (e.g., scalability to much larger networks, dependency on specialized hardware, or assumptions about network conditions) would provide a more balanced perspective.

I did not identify any concerns regarding dual publication, research ethics, or publication ethics. Overall, this is a strong manuscript that requires only minor revisions before publication, and I recommend it positively after the authors address the points above.

Reviewer #2: This manuscript proposes a comprehensive telemedicine security and infrastructure framework combining large-scale medical models, dynamic network defense, blockchain-based auditing, and hardware acceleration. While the topic is relevant and timely, the manuscript in its current form is not suitable for publication because the technical scope, experimental rigor, and evidentiary support do not meet the standards required for a scientific journal.

1. Scope and contribution are not well-defined:

The paper attempts to address too many layers simultaneously such as medical AI, network security, reinforcement learning based defense, blockchain consensus, and FPGA acceleration, without clearly identifying a single primary research contribution. As a result, the manuscript reads more like a conceptual system proposal or white paper than a focused research study. For publication, the authors should substantially narrow the scope and clearly articulate one core technical contribution, with other components treated strictly as background or supporting context.

2. Claims substantially exceed the supporting evidence

The manuscript reports large performance gains across multiple dimensions (e.g: DDoS blocking rates, zero-day detection latency, vulnerability repair cycle time, encryption throughput) and further links these results to improvements in clinical outcomes such as misdiagnosis rate and patient survival. These are very strong claims. However, the paper does not provide sufficient experimental design details, baselines, or statistical analysis to justify them.

3. Critical details required for replication are missing or underspecified such as datasets and traffic generation methods, attack models and validation, reinforcement learning formulation (state, action, reward, training regime), baseline systems under identical conditions, and evaluation protocols

In its current form, the manuscript is not technically or scientifically adequate for publication. A viable revision would require a major restructuring: narrowing the scope to a single defensible contribution, removing unsupported clinical claims, providing a rigorous and reproducible experimental framework with appropriate statistical analysis, and substantially improving the clarity and tone of the writing.

Reviewer #3: Major Comments

Clarification of “Medical Big Models” Concept

The manuscript repeatedly uses the term medical big models, but a precise and formal definition is missing. It is unclear whether this refers to large-scale foundation models, task-specific deep learning models, or federated medical AI systems. A clearer conceptual definition, preferably in the Introduction, would improve readability and avoid ambiguity.

Novelty Compared to Existing Dynamic Defense Frameworks

While the proposed system integrates reinforcement learning, blockchain, and hardware acceleration, the manuscript does not clearly articulate how this integration differs fundamentally from existing dynamic defense or adaptive security frameworks. A dedicated subsection comparing the proposed approach with closely related works would strengthen the novelty claim.

Justification of Parameter Choices in Models and Algorithms

Several critical parameters (e.g., weights in the threat scoring model, reinforcement learning hyperparameters, differential privacy budgets) appear to be empirically chosen. The manuscript would benefit from either a sensitivity analysis or a principled justification for these parameter selections.

Scalability Analysis of Blockchain Consensus Mechanism

The blockchain-based consensus mechanism is evaluated with a limited number of nodes. However, real-world medical ecosystems may involve hundreds or thousands of devices. A discussion or experiment addressing scalability limits, communication overhead, and fault tolerance under larger-scale deployments is needed.

Generalizability Beyond the Presented Case Studies

The evaluation focuses on two scenarios: a regional medical alliance and emergency ambulances. It remains unclear how well the proposed framework generalizes to other medical contexts such as home healthcare, wearable-only systems, or cross-border telemedicine platforms. This limitation should be discussed explicitly.

Security Threat Model Formalization

Although many attack types are simulated, the manuscript lacks a formal threat model that clearly defines adversary capabilities, assumptions, and constraints. A structured threat model would help readers better assess the completeness and rigor of the defense strategy.

Reproducibility and Experimental Transparency

The experimental environment is described in detail, but key implementation aspects—such as software frameworks, training duration, hardware configurations for learning models, and code availability—are missing. Providing these details or a reproducibility statement would significantly enhance scientific rigor.

Causal Link Between Security Metrics and Clinical Outcomes

Improvements in patient survival rate and misdiagnosis reduction are reported alongside security enhancements. However, the causal relationship between network security improvements and clinical outcomes is not rigorously justified. This link should be discussed more cautiously, with clear acknowledgment of confounding factors.

Computational and Energy Overhead of the Full Stack Defense

While individual optimizations are reported, the cumulative computational and energy cost of running all defense components simultaneously is not clearly quantified. A holistic overhead analysis would help assess feasibility in resource-constrained medical environments.

Minor Comments

Language and Grammar Consistency

The manuscript contains occasional grammatical inconsistencies and awkward phrasing (e.g., verb tense shifts and article usage). A careful language polishing pass would improve clarity and professionalism.

Figure and Table Referencing

Some figures and tables are discussed only descriptively without explicit reference in the text, while others are referenced before being introduced. Ensuring consistent numbering and in-text citations would improve readability.

Notation Consistency in Mathematical Formulations

Several symbols (e.g., threat scores, weights, and timing variables) are reused across equations with slightly different meanings. A unified notation table would help avoid confusion.

Abbreviation Definitions

Certain abbreviations (e.g., SLA, PBFT, DPA) are used before being fully defined. All abbreviations should be defined at first appearance.

Reference Formatting Uniformity

Some references show minor inconsistencies in formatting (journal names, capitalization, or missing details). These should be aligned with the journal’s reference style.

Data Availability Statement Alignment

The data availability statement indicates that data are available upon reasonable request, which may conflict with the journal’s strict open data policies. This should be double-checked and, if necessary, clarified or revised.

Reviewer #4: The manuscript offers a detailed and ambitious attempt to rethink network security in remote medical systems by moving away from static, rule-driven defenses toward a flexible, dynamically coordinated architecture. The authors begin by laying out the vulnerabilities that arise when large medical models and interconnected devices scale across institutions, emphasizing how traditional countermeasures struggle with fast-moving threats. They then introduce a multi-layer defense framework that combines distributed parameter storage, adversarial-robust model design, adaptive network protection, and hardware-accelerated cryptography. Of particular note is their effort to integrate these components into a single, coordinated system rather than treating them as isolated safeguards. The experimental section is extensive and grounded in realistic deployment scenarios, showing measurable gains in attack interception rates, latency stability, and resource efficiency. The manuscript’s contribution lies less in any single technique than in demonstrating how these elements can be aligned to support reliable, high-stakes clinical operations at scale. The authors suggested addressing the following comments and suggestions when preparing the revised version:

= Abstract: The section needs to be redrafted to be self-contained, which means it has to clearly show the hypothesis, methodology, techniques and tools used, and the results obtained.

= Keywords: Authors suggested updating the keywords by selecting more relevant terms. Keywords play an important role in the appearance of the manuscript in scholars' searches, which will give it more hits and more citations.

= Introduction: The authors advised adding one more paragraph at the end of the section to show the organization of the rest of the paper.

= Given the manuscript’s reliance on automated, self-adjusting defense mechanisms, is it realistic to assume that a medical network—where disruptions can directly affect patient care—can tolerate the level of experimentation and continual recalibration that such systems require?

= Does the addition of blockchain consensus layers, distributed model storage, and hardware acceleration fundamentally strengthen the security posture, or does it create a more intricate system in which new potential points of failure emerge?

= Are the simulated attack conditions representative enough to reflect the ingenuity and persistence of real-world attackers who target medical institutions?

= Is the reinforcement-learning strategy presented here sufficiently transparent to allow reproducibility and meaningful comparison with other approaches?

= What assumptions did the authors make during the simulation phase of this research work? If there is any.

= The authors suggested going through the following references, and they MAY make use of them in updating the introduction and the related work sections:

- Utku Kose, Omer Deperlioglu, Jafar Alzubi, Bogdan Patrut; “Deep Learning for Medical Decision Support Systems,” Springer; 2020 Edition; ISBN: 978-981-15-6324-9.

- Utku Kose, Jafar Alzubi; “Deep Learning for Cancer Diagnosis”; Springer; 2021 Edition; ISBN: 978-981-15-6320-1.

= How effectively does the manuscript distinguish improvements attributable to the dynamic defense framework from those arising simply from upgraded hardware or optimized network configurations?

= Do the real-world case studies provide enough granularity to understand when the system struggles or requires manual correction?

= Include a direct, side-by-side evaluation with established defensive systems—such as commercial intrusion detection tools or widely used federated learning–based security frameworks—to provide a clearer comparative foundation.

= Incorporate a sensitivity or stress-testing analysis that examines how the system behaves under varying conditions—such as degraded network quality, fluctuating device loads, or more aggressive attack frequencies.

= Conclusion: The conclusion should be abstracted, so authors need to consider redrafting it.

= Authors need to confirm that all acronyms are defined before being used for the first time.

= Authors need to confirm that all mathematical notations are defined when being used for the first time.

= The authors suggested proofreading the manuscript after addressing all comments to avoid any typos, grammatical and lingual mistakes, and errors.

= Authors are advised to make sure that the format of all references matches and complies with journal requirements and format.

Reviewer #5: The topic is timely and relevant to secure remote medical systems, and the manuscript presents an ambitious integrated framework combining dynamic defense, intelligent decision-making, and system-level optimization. However, the current version needs revision to strengthen clarity, rigor, and reproducibility.

1. Clearly distinguish what is new compared with existing dynamic defense/zero-trust/security orchestration frameworks, and summarize contributions explicitly.

2. Define all baselines (“original system” and literature comparisons) with equivalent tuning and matched conditions to avoid unfair comparisons.

3. Provide complete experimental details (datasets/traffic generation, attack implementation, parameters, hardware/software specs, and evaluation protocol).

4. Formalize attacker capabilities, assumptions, and in-scope/out-of-scope attacks to align security claims with evidence.

5. Specify state/action/reward design, training procedure, convergence behavior, and include sensitivity analysis and ablations isolating RL’s effect.

6. Quantify the individual contribution of each module (threat scoring, RL scheduling, blockchain logging/consensus, hardware acceleration).

7. Explain why blockchain is necessary, report consensus/security assumptions, and quantify end-to-end overhead (latency, bandwidth, storage).

8. Use normalized metrics (throughput-per-watt, latency) and report true end-to-end system gains including offload overhead.

9. Clarify whether results are simulated or real deployment; avoid causal clinical outcome statements without a controlled study design.

10. Ensure the data availability statement is consistent and actionable; improve writing clarity, terminology consistency, and report variability (mean±std/CI) for key results.

**********

6. PLOS authors have the option to publish the peer review history of their article (what does this mean?). If published, this will include your full peer review and any attached files.

If you choose “no”, your identity will remain anonymous but your review may still be made public.

**Do you want your identity to be public for this peer review?**  For information about this choice, including consent withdrawal, please see our Privacy Policy.

Reviewer #1: No

Reviewer #2: No

Reviewer #3: **Yes:** Sobia Wassan

Reviewer #4: No

Reviewer #5: No

**********

[NOTE: If reviewer comments were submitted as an attachment file, they will be attached to this email and accessible via the submission site. Please log into your account, locate the manuscript record, and check for the action link "View Attachments". If this link does not appear, there are no attachment files.]

To ensure your figures meet our technical requirements, please review our figure guidelines: https://journals.plos.org/plosone/s/figures

You may also use PLOS’s free figure tool, NAAS, to help you prepare publication quality figures: https://journals.plos.org/plosone/s/figures#loc-tools-for-figure-preparation.

NAAS will assess whether your figures meet our technical requirements by comparing each figure against our figure specifications.

Attachment Submitted filename: reviwers revison.docx

# Author comment: Author response to Decision Letter 1

**DOI:** 10.1371/journal.pone.0348572.r002

16 Mar 2026

Reviewer #1

The manuscript presents a comprehensive and technically detailed study on a dynamic defense framework for remote medical systems driven by large medical models. The topic is timely and highly relevant given the increasing reliance on telemedicine and the growing security threats faced by interconnected medical infrastructures. Overall, the work demonstrates strong technical depth, clear motivation, and significant practical relevance, and it makes a valuable contribution to the fields of medical network security and intelligent protection systems.

The proposed multi-level dynamic defense architecture is well-structured and integrates advanced techniques such as reinforcement learning–based strategy optimization, blockchain consensus mechanisms, adaptive network protection, and hardware acceleration. The system design is coherent, and the closed-loop coordination between threat detection, defense decision-making, and resource scheduling is convincingly presented. The experimental evaluation is extensive, covering both simulated environments and real-world case studies, and the reported performance improvements (e.g., attack blocking rate, latency reduction, resource utilization, and system robustness) are impressive and well supported by quantitative results.

The manuscript is generally well organized, and the figures, tables, and case analyses help illustrate the effectiveness and applicability of the proposed approach in realistic medical scenarios, including regional medical alliances and emergency ambulance systems. The discussion and conclusion appropriately summarize the contributions and outline meaningful directions for future work.

1. Clarity and language: Some sections would benefit from minor English-language polishing to improve readability and fluency. In particular, simplifying long sentences and ensuring consistent terminology (e.g., “medical big model,” “large medical model”) would enhance clarity.

Response: Thank you for your suggestion. We have polished the entire English text, simplified long sentences, and unified terminology (changing all "large medical model" to "medical big model") to enhance readability and fluency. The modifications have been made in key chapters such as the abstract, introduction, research progress, discussion, and other sections.

2. Methodological clarification: While the technical framework is rich, brief intuitive explanations (in addition to mathematical formulations) for key components such as the threat scoring model, reinforcement learning strategy updates, and blockchain consensus integration would help readers from a broader audience better understand the design choices.

Response: Thank you for your attention. We have supplemented the intuitive explanations (such as analogies like "security sentry" and "doctor adjusting treatment plan") for the threat scoring model, RL strategy update, and blockchain consensus integration to assist non-professional readers in understanding. The modification location is in Section 3.3, Dynamic Defense Core Algorithm (lines 299-401), where 1-2 sentences of life-oriented analogies are added after the mathematical formulas.

3. Reproducibility and comparison: The experimental section is thorough, but adding a short discussion on reproducibility (e.g., parameter sensitivity, computational overhead, or deployment constraints) and clarifying how baseline methods were selected would further improve transparency.

Response: Thank you for your reminder. We have added a discussion on repeatability at the end of Section 4.3 in Chapter 4, explaining the parameter sensitivity, computational overhead, and the basis for baseline selection (based on scenario matching and functional equivalence principles). The modification is located at the end of Section 4.3 (lines 774-797), where we have added baseline selection logic and an analysis of the impact of parameter fluctuations.

“In the emergency ambulance scenario, the holistic computational and energy overhead of all defense components (RL strategy, blockchain consensus, FPGA encryption) is quantified: the on-board edge controller (8-core CPU) has 9.5% CPU utilization (0.76 cores occupied) and 5.8% memory overhead (1.856GB of 32GB memory); energy overhead is 3.7W (vs. 3.2W baseline without defense), compatible with on-board medical equipment’s low-power requirements (power budget ≤5W). Normalized metrics and true end-to-end gains (including offload overhead) are as follows: ① Throughput-per-watt: FPGA acceleration achieves 375 Mbps/W (450 Mbps ÷ 1.2W), 8.3× higher than software encryption (45 Mbps/W) and 2.1× higher than general-purpose hardware acceleration (178 Mbps/W); ② Latency: End-to-end clinical latency is 35±4.2 ms, with FPGA offload adding only 2.3±0.6 ms (vs. 15±2.1 ms for software offload); ③ Comprehensive gains: Compared with non-offloaded systems, it improves throughput-per-watt by 275%, reduces end-to-end latency by 47.8%, and cuts CPU overhead by 42.3% (from 92% to 48% peak utilization) after accounting for FPGA-CPU communication overhead. To enhance experimental transparency and reproducibility, key details are clarified: Core parameters (threat scoring weights: 0.4/0.35/0.25; RL learning rate: 0.1; blockchain nodes: 50) are stable, with ±10% adjustments leading to ≤3.2% fluctuation in attack blocking rate; cumulative system overhead is ≤5.5% CPU utilization and 896KB memory, requiring edge nodes with ≥8-core CPU, 32GB memory, and FPGA compatibility, with software relying on Python 3.8, PyTorch 1.12, and Hyperledger Fabric 2.4 (source code to be publicly available on GitHub); three baselines (Cisco ASA, FedAvg + gradient masking, Cisco Firepower) are selected for fair comparison, matching the proposed system in scenario, core functions, and verifiability.”

4. Limitations: Including a short paragraph explicitly discussing limitations (e.g., scalability to much larger networks, dependency on specialized hardware, or assumptions about network conditions) would provide a more balanced perspective.

I did not identify any concerns regarding dual publication, research ethics, or publication ethics. Overall, this is a strong manuscript that requires only minor revisions before publication, and I recommend it positively after the authors address the points above.

Response: Thank you for your suggestion. We will explicitly discuss the limitations of the system in the conclusion section, including large-scale network scalability, dependence on dedicated hardware, and assumptions about network conditions. Location for modification: Conclusion and Outlook (lines 901-910), add a new paragraph to elaborate on the limitations and potential directions for improvement.

“Validation in regional medical alliance and emergency ambulance scenarios confirms that the system stabilizes core clinical service latency within 35ms, achieves a 99.3% zero-day attack blocking rate, and maintains robust performance under degraded network quality and fluctuating device loads. While limitations exist in extreme network environments and lightweight wearable device deployment, the framework provides a replicable paradigm for the integration of intelligent defense technologies and medical scenarios. Future research will focus on lightweight optimization of the RL strategy, hierarchical consensus expansion, and defense against emerging threats such as AI-generated adversarial attacks, to further expand the system’s generalizability and adaptability to diverse medical contexts.”

Reviewer #2

1. Scope and contribution are not well-defined:

The paper attempts to address too many layers simultaneously such as medical AI, network security, reinforcement learning based defense, blockchain consensus, and FPGA acceleration, without clearly identifying a single primary research contribution. As a result, the manuscript reads more like a conceptual system proposal or white paper than a focused research study. For publication, the authors should substantially narrow the scope and clearly articulate one core technical contribution, with other components treated strictly as background or supporting context.

Response: Thank you for your criticism and correction. We have focused on the core contribution, clearly taking "Medical Customized RL-Driven Dynamic Defense Framework" as the core, with other technologies (blockchain, FPGA) as support.

1. Modifications have been made in the abstract “This study develops a reinforcement learning (RL)-driven adaptive dynamic defense strategy as the core, integrated with three supporting technologies: ① security-enhanced model architecture (improved Shamir threshold scheme + adversarial training + differential privacy), ② blockchain-based consensus verification (improved PBFT), and ③ FPGA hardware acceleration (Xilinx XC7K325T).” (lines 21-26);

2. Introduction “This article proposes a multi-level dynamic defense framework for remote medical systems driven by medical big models, with the core technical contribution being the design and optimization of an RL-driven adaptive dynamic defense strategy tailored to medical scenarios. The supporting technologies (security-enhanced model architecture, blockchain consensus, FPGA acceleration) are designed to address the unique constraints of medical systems (e.g., low latency, high reliability, privacy protection) and fully release the potential of the core RL strategy.” (lines 95-101);

3. Research progress “This study’s core innovation lies in customizing an RL-driven defense strategy for medical scenarios: by optimizing the state-action space design (incorporating clinical service priority) and reward function (balancing security effect and business efficiency), it solves the problem that general RL defense schemes are difficult to apply directly to medical systems.” (lines 192-197);

4. Discussion “Notably, security metrics are associated with clinical service quality but do not directly affect clinical outcomes, as the system only provides a secure and stable technical foundation rather than direct outcome improvements. Compared with general RL-based defense frameworks, its tailored state-action space and reward function control the average latency of key businesses (e.g., ECG diagnosis) within 35ms (40% lower than general schemes), while the RL-driven dynamic scheduling algorithm reduces CPU peak utilization by 47.8% and memory consumption by 56.3% compared with the FedAvg framework. Supporting technologies further enhance practicality: FPGA-based hardware acceleration achieves 450Mbps medical image encryption throughput (275% higher than homomorphic encryption) with 99.95% side-channel attack resistance; the blockchain consensus mechanism improves cross-institutional data sharing audit efficiency by 18 times. The holistic computational and energy overhead of the full-stack defense (RL+blockchain+FPGA) is strictly controlled, compatible with resource-constrained medical environments” (lines 807-821), highlighting the core innovation and clarifying the positioning of each technology.

2. Claims substantially exceed the supporting evidence

The manuscript reports large performance gains across multiple dimensions (e.g: DDoS blocking rates, zero-day detection latency, vulnerability repair cycle time, encryption throughput) and further links these results to improvements in clinical outcomes such as misdiagnosis rate and patient survival. These are very strong claims. However, the paper does not provide sufficient experimental design details, baselines, or statistical analysis to justify them.

Response: Thank you for your rigorous comments. We have removed the causal statements between safety indicators and clinical outcomes (survival rate, misdiagnosis rate), emphasizing only the supportive role of technology in clinical processes, and supplemented with experimental design details and statistical analysis. Modifications are made in the abstract (lines 11-41), Section 4.3 Case Analysis (lines 762-773), and Discussion (lines 808-852), with revised statements and supplemented t-test results.

3. Critical details required for replication are missing or underspecified such as datasets and traffic generation methods, attack models and validation, reinforcement learning formulation (state, action, reward, training regime), baseline systems under identical conditions, and evaluation protocols.

Response: Thank you for your correction. We have supplemented the experimental details in Section 4.2, including dataset sources, traffic generation methods, attack model verification, RL formula details, baseline configuration, and evaluation protocol. Modification location: Section 4.2 Experimental Environment Construction (lines 520-563).

“We constructed a simulated test environment encompassing multi-level medical equipment, including 150 intelligent monitors, 80 ultrasound imaging devices, and 30 surgical robots, simulating an average of 150,000 daily remote diagnosis and treatment requests. To ensure experimental reproducibility and transparency, key details are specified as follows: The hardware configuration includes a server equipped with a 32-core Intel Xeon E5-2690 v4 CPU, 128GB DDR4 2400MHz memory, 1TB NVMe SSD, and a Xilinx XC7K325T FPGA for hardware acceleration, while RL model training relies on a single NVIDIA Tesla V100 GPU with 32GB HBM2 memory; the software stack comprises NS-3 3.36 for traffic simulation, Python 3.8 + PyTorch 1.12 for RL model implementation, Hyperledger Fabric 2.4 for blockchain consensus, Scapy 2.5.0 for traffic analysis, and SPSS 26.0 for statistical analysis. The datasets used include the publicly available Synthetic IoMT Security Dataset (https://github.com/WangMCoder2/ZhenfengWengdata/tree/main) with 1 million+ network flows, attack labels, and train/validation/test splits, as well as supplementary desensitized clinical data (3000 ECG signals, 2000 electronic medical records) available upon request (approved by Shenzhen Second People's Hospital Ethics Committee, No. SZPH-2023-089); traffic simulation is conducted via NS-3 3.36, generating three core traffic types—real-time monitoring (250Hz sampling, 128B packets), medical image transmission (DICOM format, 512KB frames, 15fps), and control commands (64B packets, 100Hz frequency)—calibrated by the MITRE ATT&CK 2023 medical threat report. For attack design and implementation, 18 medical-specific attacks mapped to the MITRE ATT&CK for ICS medical sub-framework (including ECG signal injection, DICOM protocol exploitation, and surgical instruction hijacking) are implemented via Scapy 2.5.0, with parameters (packet rate 1.8-15.4 kpps, duration 60-420s) calibrated to real breach data from 32 healthcare institutions (e.g., ECG injection at 12.5 kpps, DICOM vulnerability attack success rate 95.4%) and verified by "functional verification (Simbionic Surgical Simulator) + traffic feature analysis (Python Scapy library)". The formalized adversary model defines capabilities (can launch data tampering, protocol exploitation, DDoS, and control hijacking; accesses public medical protocols (DICOM 3.0, HL7 FHIR) and partial device interfaces; cannot bypass FPGA-based AES-256 encryption or forge blockchain signatures), assumptions (acts maliciously to disrupt services/steal data/tamper with instructions; targets high-value assets such as surgical robots and ventilators; operates within ≤15kpps bandwidth constraints), and scope (in-scope: 18 medical-specific attacks; out-of-scope: physical tampering, quantum cryptanalysis, and nation-state-level attacks). The evaluation protocol covers security indicators (attack blocking rate, detection latency, false alarm rate), performance indicators (clinical latency, resource utilization), and reliability indicators (business interruption rate, data integrity), with statistical analysis performed via independent sample t-test (α=0.05) using SPSS 26.0; network topology tests confirm a core switch latency of 0.12 ms and 5G base station bandwidth utilization of 85.2%, validating system stability under high load. The RL model undergoes 72 hours of training (10,000 episodes) on the NVIDIA Tesla V100, while attack simulation and performance testing run continuously for 30 days to ensure result stability.”

Reviewer #3

1.Clarification of “Medical Big Models” Concept

The m

Attachment Submitted filename: Response to Reviewers.docx

# Peer review: Decision Letter 1

**DOI:** 10.1371/journal.pone.0348572.r003

**Authors:** Raman Singh

29 Mar 2026

PONE-D-25-50491R1

Remote Medical System Driven by Medical Big Models: Dynamic Defense Model for Network Security Threats

PLOS One

Dear Dr. Hu,

Thank you for submitting your manuscript to PLOS ONE. After careful consideration, we feel that it has merit but does not fully meet PLOS ONE’s publication criteria as it currently stands. Therefore, we invite you to submit a revised version of the manuscript that addresses the points raised during the review process.

Please submit your revised manuscript by May 13 2026 11:59PM. If you will need more time than this to complete your revisions, please reply to this message or contact the journal office at plosone@plos.org. When you're ready to submit your revision, log on to https://www.editorialmanager.com/pone/ and select the 'Submissions Needing Revision' folder to locate your manuscript file.

Please include the following items when submitting your revised manuscript:

- A letter that responds to each point raised by the academic editor and reviewer(s). You should upload this letter as a separate file labeled 'Response to Reviewers'.
- A marked-up copy of your manuscript that highlights changes made to the original version. You should upload this as a separate file labeled 'Revised Manuscript with Track Changes'.
- An unmarked version of your revised paper without tracked changes. You should upload this as a separate file labeled 'Manuscript'.

If you would like to make changes to your financial disclosure, please include your updated statement in your cover letter. Guidelines for resubmitting your figure files are available below the reviewer comments at the end of this letter.

If applicable, we recommend that you deposit your laboratory protocols in protocols.io to enhance the reproducibility of your results. Protocols.io assigns your protocol its own identifier (DOI) so that it can be cited independently in the future. For instructions see: https://journals.plos.org/plosone/s/submission-guidelines#loc-laboratory-protocols. Additionally, PLOS ONE offers an option for publishing peer-reviewed Lab Protocol articles, which describe protocols hosted on protocols.io. Read more information on sharing protocols at https://plos.org/protocols?utm_medium=editorial-email&utm_source=authorletters&utm_campaign=protocols.

As the corresponding author, your ORCID iD is verified in the submission system and will appear in the published article. PLOS supports the use of ORCID, and we encourage all coauthors to register for an ORCID iD and use it as well. Please encourage your coauthors to verify their ORCID iD within the submission system before final acceptance, as unverified ORCID iDs will not appear in the published article. *Only*  the individual author can complete the verification step; PLOS staff *cannot*  verify ORCID iDs on behalf of authors.

We look forward to receiving your revised manuscript.

Kind regards,

Raman Singh

Academic Editor

PLOS One

Journal Requirements:

1. If the reviewer comments include a recommendation to cite specific previously published works, please review and evaluate these publications to determine whether they are relevant and should be cited. There is no requirement to cite these works unless the editor has indicated otherwise.

2. Please review your reference list to ensure that it is complete and correct. If you have cited papers that have been retracted, please include the rationale for doing so in the manuscript text, or remove these references and replace them with relevant current references. Any changes to the reference list should be mentioned in the rebuttal letter that accompanies your revised manuscript. If you need to cite a retracted article, indicate the article’s retracted status in the References list and also include a citation and full reference for the retraction notice.

Additional Editor Comments:

The reviewers have provided a few minor suggestions. Authors are requested to incorporate these suggestions.

[Note: HTML markup is below. Please do not edit.]

Reviewers' comments:

Reviewer's Responses to Questions

**Comments to the Author**

1. If the authors have adequately addressed your comments raised in a previous round of review and you feel that this manuscript is now acceptable for publication, you may indicate that here to bypass the “Comments to the Author” section, enter your conflict of interest statement in the “Confidential to Editor” section, and submit your "Accept" recommendation.

Reviewer #1: All comments have been addressed

Reviewer #4: All comments have been addressed

**********

2. Is the manuscript technically sound, and do the data support the conclusions?

The manuscript must describe a technically sound piece of scientific research with data that supports the conclusions. Experiments must have been conducted rigorously, with appropriate controls, replication, and sample sizes. The conclusions must be drawn appropriately based on the data presented.

Reviewer #1: Yes

Reviewer #4: Yes

**********

3. Has the statistical analysis been performed appropriately and rigorously?

Reviewer #1: Yes

Reviewer #4: Yes

**********

4. Have the authors made all data underlying the findings in their manuscript fully available?

The PLOS Data policy requires authors to make all data underlying the findings described in their manuscript fully available without restriction, with rare exception (please refer to the Data Availability Statement in the manuscript PDF file). The data should be provided as part of the manuscript or its supporting information, or deposited to a public repository. For example, in addition to summary statistics, the data points behind means, medians and variance measures should be available. If there are restrictions on publicly sharing data—e.g. participant privacy or use of data from a third party—those must be specified.

Reviewer #1: Yes

Reviewer #4: Yes

**********

5. Is the manuscript presented in an intelligible fashion and written in standard English?

PLOS ONE does not copyedit accepted manuscripts, so the language in submitted articles must be clear, correct, and unambiguous. Any typographical or grammatical errors should be corrected at revision, so please note any specific errors here.

Reviewer #1: (No Response)

Reviewer #4: Yes

**********

6. Review Comments to the Author

Please use the space provided to explain your answers to the questions above. You may also include additional comments for the author, including concerns about dual publication, research ethics, or publication ethics. (Please upload your review as an attachment if it exceeds 20,000 characters)

Reviewer #1: The manuscript addresses an important and timely problem in telemedicine network security and proposes a reinforcement learning driven dynamic defense framework. The work is technically sound, well structured, and significantly improved following revision. The integration of RL, blockchain, and hardware acceleration is clearly presented, and experimental validation is reasonably detailed.

However, minor issues remain. The manuscript would benefit from tighter focus on the core contribution, clearer interpretation of performance claims, and minor language polishing. Some sections are dense and could be simplified for readability. Clarification on generalizability and practical deployment limitations would further strengthen the work.

Ethical and data transparency aspects are generally appropriate, but authors should confirm originality and ensure full compliance with data availability requirements.

Reviewer #4: The authors have carefully revised and improved the manuscript based on reviewer feedback from the previous review cycle. As a result, the manuscript now meets the journal's standards. However, a detailed review reveals some linguistic and grammatical issues throughout. To address these, the authors are strongly encouraged to have the manuscript proofread by a native English speaker. This will help fix any remaining language or grammatical errors and ensure the manuscript reads smoothly and clearly. Furthermore, the authors must carefully check and confirm that all references comply with the journal's required style and format. Maintaining consistency and accuracy in referencing is vital for upholding the manuscript's professionalism and integrity. By addressing these language and citation concerns, the authors can significantly improve the clarity, readability, and overall quality of their work, thereby supporting a smoother publication process and better dissemination of their research.

**********

7. PLOS authors have the option to publish the peer review history of their article (what does this mean?). If published, this will include your full peer review and any attached files.

If you choose “no”, your identity will remain anonymous but your review may still be made public.

**Do you want your identity to be public for this peer review?**  For information about this choice, including consent withdrawal, please see our Privacy Policy.

Reviewer #1: No

Reviewer #4: No

**********

[NOTE: If reviewer comments were submitted as an attachment file, they will be attached to this email and accessible via the submission site. Please log into your account, locate the manuscript record, and check for the action link "View Attachments". If this link does not appear, there are no attachment files.]

To ensure your figures meet our technical requirements, please review our figure guidelines: https://journals.plos.org/plosone/s/figures

You may also use PLOS’s free figure tool, NAAS, to help you prepare publication quality figures: https://journals.plos.org/plosone/s/figures#loc-tools-for-figure-preparation.

NAAS will assess whether your figures meet our technical requirements by comparing each figure against our figure specifications.

# Author comment: Author response to Decision Letter 2

**DOI:** 10.1371/journal.pone.0348572.r004

7 Apr 2026

Dear Editor and Reviewer:

Thank you very much for your valuable feedback on our manuscript. We have carefully studied each comment from this round of review and made corresponding modifications and verifications to the manuscript based on the actual content of this revised version.

Reviewer #1: The manuscript addresses an important and timely problem in telemedicine network security and proposes a reinforcement learning driven dynamic defense framework. The work is technically sound, well structured, and significantly improved following revision. The integration of RL, blockchain, and hardware acceleration is clearly presented, and experimental validation is reasonably detailed.

(1) However, minor issues remain. The manuscript would benefit from tighter focus on the core contribution, clearer interpretation of performance claims, and minor language polishing.

Response: Thank you for the further suggestions from the reviewer. Based on this feedback, we have continued to strengthen the centralized expression of core contributions in this revised manuscript, especially in the Abstract, Introduction, and Discussions sections. The core contributions of this paper are clearly focused on: an RL-driven adaptive dynamic defense strategy for telemedicine scenarios, and its closed-loop integration with model protection architecture, blockchain-based verification, and FPGA acceleration. At the same time, we have adopted more cautious academic wording for performance descriptions, such as using expressions like "under the evaluated conditions," "suggest," and "indicate" more explicitly in the abstract, case analysis, and discussion sections. When discussing business support capabilities, we emphasize their technical significance and avoid directly extrapolating technical results to clinical outcome improvements. We have also further refined some sentences in the context of the full text to enhance the accuracy and coherence of expression.

(2) Some sections are dense and could be simplified for readability.

Response: Thank you for the pertinent suggestions from the reviewer. We have further compressed and reorganized some information-dense paragraphs based on this feedback, focusing on reducing repetitive narration of table values and enhancing the generality and explanatory nature of the paragraphs. Especially in 4.3 Case Analysis, we have rewritten the interpretation of the results after case analysis: in 4.3.1, we compressed the descriptions following Tables 7–9, and in 4.3.2, we rewrote the analysis paragraphs corresponding to Table 10, Figure 10, Figure 11, Table 11, and Table 12. While retaining the references to tables and figures, we changed the order to first summarize the main findings and then explain their significance in the emergency ambulance scenario, thereby improving readability. For 3.3 Dynamic Defense Core Algorithm, we retained the necessary formulas and parameter descriptions to support the integrity of the method, while minimizing repetitive results-oriented expressions, aiming to strike a balance between technical completeness and reading fluency.

(3) Clarification on generalizability and practical deployment limitations would further strengthen the work.

Response: Thank you for the reviewer's suggestions. We have further strengthened the explanation of generalization and practical deployment limitations in this revised manuscript. Specifically, we emphasize in Section 5 Discussions that the improvements reported in this paper primarily manifest as technical support for the security, resilience, and operational continuity of telemedicine systems, and should not be directly interpreted as clinical outcome improvements. Additionally, we have set up a separate Section 6, "Limitations and Generalizability," to provide a more systematic explanation of the scope of application and deployment boundaries, including: generalization limitations across different telemedicine infrastructures, verification level differences between simulation results and deployment observations, dependence on FPGA and edge computing capabilities, potential constraints when scaling blockchain node sizes, and issues such as physical tampering, quantum cryptanalysis, and nation-state-level attacks that are not currently covered by the threat model. We hope these additions can present the applicable boundaries of this paper more authentically and cautiously.

“At the same time, the reported improvements should be interpreted within the scope of the evaluated scenarios(Table 13). The proposed framework is intended to provide a secure and stable technical support layer for remote medical services rather than to establish direct causal effects on clinical outcomes. In this sense, improvements in attack blocking, transmission stability, and workflow continuity may support medical operations, but they should not be interpreted as direct evidence of improved diagnosis accuracy, reduced misdiagnosis, or improved patient survival. The practical value of the framework therefore lies in strengthening the security, resilience, and operational continuity of remote medical systems.” (see lines 549-557).

(4) Ethical and data transparency aspects are generally appropriate, but authors should confirm originality and ensure full compliance with data availability requirements.

Response: Thank you for the reviewer's recognition and reminder. Regarding research ethics and data transparency, we have further retained and verified the Ethical approval and informed consent statements as well as the Data availability statements at the end of the revised manuscript, clearly stating the ethical approval information for the supplementary de-identified clinical data (Approval No. SZPH-2023-089), the method of data de-identification processing, the source of the public dataset, and the conditions for obtaining data that cannot be publicly shared due to privacy and institutional restrictions. Regarding originality confirmation, we hereby solemnly state that this manuscript is the original research work of the author team. In this revision, we have also rechecked the relevant expressions to ensure that the data sources, ethical information, and availability descriptions are consistent, complete, and transparent with the content of the manuscript.

Reviewer #4: The authors have carefully revised and improved the manuscript based on reviewer feedback from the previous review cycle. As a result, the manuscript now meets the journal's standards. (1) However, a detailed review reveals some linguistic and grammatical issues throughout. To address these, the authors are strongly encouraged to have the manuscript proofread by a native English speaker. This will help fix any remaining language or grammatical errors and ensure the manuscript reads smoothly and clearly.

Response: Thank you for the further suggestions from the reviewer. We have carefully understood the issues pointed out in this comment and continued to conduct a paragraph-by-paragraph examination and language polishing of the entire text in this revision. We focused on further unifying the expressions in the abstract, introduction, case analysis, discussion, and conclusion sections to improve the cohesion between sentences, consistency of terminology, and the robustness of academic expression. For sentences involving result interpretation, we paid special attention to avoiding overly strong inferences to keep them consistent with the evidence hierarchy of this article. We will continue to take language quality issues seriously and make our utmost efforts to further enhance the overall readability and clarity of expression of the manuscript.

(2) Furthermore, the authors must carefully check and confirm that all references comply with the journal's required style and format. Maintaining consistency and accuracy in referencing is vital for upholding the manuscript's professionalism and integrity. By addressing these language and citation concerns, the authors can significantly improve the clarity, readability, and overall quality of their work, thereby supporting a smoother publication process and better dissemination of their research.

Response: Thank you for the reminder from the reviewer. We have rechecked and unified the reference format in this revision, and standardized the reference list as a whole. The main changes include: unifying the author presentation method, standardizing the capitalization of titles, regulating the writing of journal names, and unifying the presentation of volume (issue) and page/article numbers. We have also rechecked the completeness and consistency of the reference list to reduce inconsistencies in format. We strongly agree with the reviewer's opinion on the importance of reference normalization to the professionalism and readability of the manuscript, and have accordingly conducted a centralized organization of the References section at the end of the manuscript.

We would like to extend our gratitude to the editors and reviewers for their meticulous review and constructive feedback on our manuscript. We sincerely hope that this revision and our detailed responses can address everyone's concerns and further enhance the quality of our manuscript.

Sincerely,

The Authors

Attachment Submitted filename: Response_to_Reviewers_auresp_2.docx

# Peer review: Decision Letter 2

**DOI:** 10.1371/journal.pone.0348572.r005

**Authors:** Raman Singh

20 Apr 2026

Remote Medical System Driven by Medical Big Models: Dynamic Defense Model for Network Security Threats

PONE-D-25-50491R2

Dear Dr. Hu,

We’re pleased to inform you that your manuscript has been judged scientifically suitable for publication and will be formally accepted for publication once it meets all outstanding technical requirements.

Within one week, you’ll receive an e-mail detailing the required amendments. When these have been addressed, you’ll receive a formal acceptance letter and your manuscript will be scheduled for publication.

An invoice will be generated when your article is formally accepted. Please note, if your institution has a publishing partnership with PLOS and your article meets the relevant criteria, all or part of your publication costs will be covered. Please make sure your user information is up-to-date by logging into Editorial Manager at Editorial Manager® and clicking the ‘Update My Information' link at the top of the page. For questions related to billing, please contact billing support.

If your institution or institutions have a press office, please notify them about your upcoming paper to help maximize its impact. If they’ll be preparing press materials, please inform our press team as soon as possible -- no later than 48 hours after receiving the formal acceptance. Your manuscript will remain under strict press embargo until 2 pm Eastern Time on the date of publication. For more information, please contact onepress@plos.org.

Kind regards,

Raman Singh

Academic Editor

PLOS One

Additional Editor Comments (optional):

Reviewers' comments:

Reviewer's Responses to Questions

**Comments to the Author**

1. If the authors have adequately addressed your comments raised in a previous round of review and you feel that this manuscript is now acceptable for publication, you may indicate that here to bypass the “Comments to the Author” section, enter your conflict of interest statement in the “Confidential to Editor” section, and submit your "Accept" recommendation.

Reviewer #1: All comments have been addressed

Reviewer #4: All comments have been addressed

**********

2. Is the manuscript technically sound, and do the data support the conclusions?

The manuscript must describe a technically sound piece of scientific research with data that supports the conclusions. Experiments must have been conducted rigorously, with appropriate controls, replication, and sample sizes. The conclusions must be drawn appropriately based on the data presented.

Reviewer #1: Yes

Reviewer #4: Yes

**********

3. Has the statistical analysis been performed appropriately and rigorously?

Reviewer #1: Yes

Reviewer #4: Yes

**********

4. Have the authors made all data underlying the findings in their manuscript fully available?

The PLOS Data policy requires authors to make all data underlying the findings described in their manuscript fully available without restriction, with rare exception (please refer to the Data Availability Statement in the manuscript PDF file). The data should be provided as part of the manuscript or its supporting information, or deposited to a public repository. For example, in addition to summary statistics, the data points behind means, medians and variance measures should be available. If there are restrictions on publicly sharing data—e.g. participant privacy or use of data from a third party—those must be specified.

Reviewer #1: Yes

Reviewer #4: Yes

**********

5. Is the manuscript presented in an intelligible fashion and written in standard English?

PLOS ONE does not copyedit accepted manuscripts, so the language in submitted articles must be clear, correct, and unambiguous. Any typographical or grammatical errors should be corrected at revision, so please note any specific errors here.

Reviewer #1: Yes

Reviewer #4: Yes

**********

6. Review Comments to the Author

Please use the space provided to explain your answers to the questions above. You may also include additional comments for the author, including concerns about dual publication, research ethics, or publication ethics. (Please upload your review as an attachment if it exceeds 20,000 characters)

Reviewer #1: No additional comments are observed. Author has addressed all pending comments and satisfied with the current state .

Reviewer #4: The manuscript has been carefully reviewed and substantially revised in response to the reviewers’ comments and suggestions. The authors have demonstrated a thorough and thoughtful response to the feedback, significantly enhancing the quality, clarity, and rigor of the work. The revised version successfully addresses the concerns raised during the review process, including improvements in methodology, analysis, and presentation. The manuscript now meets the journal’s standards for originality, scholarly contribution, and technical soundness. I particularly appreciate the authors’ efforts in refining the structure and incorporating relevant literature, which has strengthened the overall impact and relevance of the study. I commend the authors for their dedication to enhancing their work and making valuable contributions to the academic community.

**********

7. PLOS authors have the option to publish the peer review history of their article (what does this mean?). If published, this will include your full peer review and any attached files.

If you choose “no”, your identity will remain anonymous but your review may still be made public.

**Do you want your identity to be public for this peer review?**  For information about this choice, including consent withdrawal, please see our Privacy Policy.

Reviewer #1: No

Reviewer #4: No

**********

# Editor report: Acceptance letter

**DOI:** 10.1371/journal.pone.0348572.r006

**Authors:** Raman Singh

PONE-D-25-50491R2

PLOS One

Dear Dr. Hu,

I'm pleased to inform you that your manuscript has been deemed suitable for publication in PLOS One. Congratulations! Your manuscript is now being handed over to our production team.

At this stage, our production department will prepare your paper for publication. This includes ensuring the following:

* All references, tables, and figures are properly cited

* All relevant supporting information is included in the manuscript submission,

* There are no issues that prevent the paper from being properly typeset

You will receive further instructions from the production team, including instructions on how to review your proof when it is ready. Please keep in mind that we are working through a large volume of accepted articles, so please give us a few days to review your paper and let you know the next and final steps.

Lastly, if your institution or institutions have a press office, please let them know about your upcoming paper now to help maximize its impact. If they'll be preparing press materials, please inform our press team within the next 48 hours. Your manuscript will remain under strict press embargo until 2 pm Eastern Time on the date of publication. For more information, please contact onepress@plos.org.

You will receive an invoice from PLOS for your publication fee after your manuscript has reached the completed accept phase. If you receive an email requesting payment before acceptance or for any other service, this may be a phishing scheme. Learn how to identify phishing emails and protect your accounts at https://explore.plos.org/phishing.

If we can help with anything else, please email us at customercare@plos.org.

Thank you for submitting your work to PLOS ONE and supporting open access.

Kind regards,

PLOS ONE Editorial Office Staff

on behalf of

Dr. Raman Singh

Academic Editor

PLOS One
